# Duration of prone position sessions: a prospective cohort study

**DOI:** 10.1186/s13613-020-00683-7

**Published:** 2020-05-24

**Authors:** Sebastien Jochmans, Sandie Mazerand, Jonathan Chelly, Franck Pourcine, Oumar Sy, Nathalie Thieulot-Rolin, Olivier Ellrodt, Emmanuelle Mercier Des Rochettes, Gaël Michaud, Jean Serbource-Goguel, Christophe Vinsonneau, Ly Van Phach Vong, Mehran Monchi

**Affiliations:** 1 Département de Médecine Intensive-Réanimation, GH Sud Ile-de-France, Hôpital de Melun, 270 avenue Marc Jacquet, 77000 Melun, France; 2 Unité de Recherche Clinique, GH Sud Ile-de-France, Hôpital de Melun, 270 avenue Marc Jacquet, 77000 Melun, France; 3 Service de Réanimation Médicale, AP-HP, Hôpital Saint-Antoine, 184 rue du Faubourg Saint-Antoine, 75012 Paris, France; 4 Service de Réanimation, Hôpital de Béthune, 27 rue Delbecque, 62660 Beuvry, France

**Keywords:** Prone position, Acute respiratory distress syndrome, Capnography, Protective ventilation, Mechanical ventilation, Acute respiratory failure

## Abstract

**Background:**

Prone position (PP) is highly recommended in moderate-to-severe ARDS. However, the optimal duration of PP sessions remains unclear. We searched to evaluate the time required to obtain the maximum physiological effect, and to search for parameters related to patient survival in PP.

**Methods and results:**

It was a prospective, monocentric, physiological study. We included in the study all prone-positioned patients in our ICU between June 2016 and January 2018. Pulmonary mechanics, data from volumetric capnography and arterial blood gas were recorded before prone positioning, 2 h after proning, before return to a supine position (SP) and 2 h after return to SP. Dynamic parameters were recorded before proning and every 30 min during the session until 24 h. 103 patients (ARDS 95%) were included performing 231 PP sessions with a mean length of 21.5 ± 5 h per session. They presented a significant increase in pH, static compliance and P_a_O_2_/F_i_O_2_ with a significant decrease in P_a_CO_2_, P_plat_, phase 3 slope of the volumetric capnography, P_et_CO_2_, *V*_D_/*V*_T-phy_ and Δ*P*. The beneficial physiological effects continued after 16 h of PP and at least up to 24 h in some patients. The evolution of the respiratory parameters during the first session and also during the pooled sessions did not find any predictor of response to PP, whether before, during or 2 h after the return in SP.

**Conclusions:**

PP sessions should be prolonged at least 24 h and be extended in the event that the P_a_O_2_/F_i_O_2_ ratio at 24 h remains below 150, especially since no criteria can predict which patient will benefit or not from it.

*Trial registration* The trial has been registered on 28 June 2016 in ClinicalTrials.gov (NCT 02816190) (https://clinicaltrials.gov/ct2/show/NCT02816190?term=propocap&rank=1).

## Background

Prone position (PP) has been part of respiratory management of moderate-to-severe acute respiratory distress syndrome (ARDS) for several years [1]. Randomized controlled trials have confirmed that oxygenation is improved in the PP compared to the supine position (SP) [2–6]. It could also prevent ventilator-induced lung injury (VILI). It would reduce pulmonary stress and strain by reducing the overdistension of aerated non-dependent zones while allowing recruitment of atelectatic dependent zones [7–10]. The compliance of the respiratory system would increase [11] despite a decrease in the compliance of the chest wall [12]. Lung expansion would be more homogeneous with improved ventilation/perfusion ratios and reduced VILI [13–16].

Two meta-analyses [17, 18] and then the PROSEVA trial [6] showed a beneficial effect on the outcome of moderate-to-severe ARDS with a reduction in mortality. PP is thus one of the three therapeutics to have shown a positive effect on ARDS mortality, with the reduction of the tidal volume (VT) [19] and the early use of neuromuscular blocking agents (NMBA) [20], although this is partly challenged by a recent study [21, 22]. This is why its use is highly recommended in severe ARDS [23, 24]. Its use in mild-to-moderate ARDS remains debated [25].

However, the optimal duration of PP sessions remains unclear. International recommendations are to leave the patient in PP for at least 12 h [23]. In the PROSEVA trial [6], they stayed on average 17 h, while the meta-analyses included studies with sessions lasting from 7 to 18 h.

We conducted a prospective monocentric physiological study, including all patients put in PP. The main objective was to evaluate the time sufficient to obtain the maximum improvement in several physiological respiratory parameters in the first PP session and in all PP sessions. The secondary objective was to search for physiological parameters related to patient survival in PP.

## Materials and methods

It was a prospective, monocentric, physiological study. We included in the study all prone-positioned patients in our intensive care unit (ICU) between June 2016 and January 2018. PP was indicated in case of moderate-to-severe hypoxemia with P_a_O_2_/F_i_O_2_ < 150 despite a set PEEP of at least 10 cmH_2_O and the use of NMBA. The sessions were to last from 16 to 24 h or more. PP was not performed in case of severe hemodynamic instability (life-threatening cardiac arrhythmia, vasopressor > 2 µg/kg/min) or withdrawal of life sustaining treatments. The PP procedure was completely protocolized (protocol available in Additional file 1). The sessions could be interrupted before 16 h in case of urgent care (CT, surgery, cardiac arrest, etc.). Otherwise the return to the supine position was made after at least 16 h, if P_a_O_2_/F_i_O_2_ > 150 and if the staff was completely available. Prone position therapy was stopped when P_a_O_2_/F_i_O_2_ remained above 150 in SP. Mechanical ventilation was delivered in a controlled ventilation mode, either in volume or in pressure. Expiratory tidal volume (VT_e_) had to be adjusted between 5 and 8 mL/kg of predicted body weight. PEEP was set to achieve an end-inspiratory plateau pressure (P_plat_) between 28 and 30 cmH_2_O (or more if an esophageal pressure probe was used to titrate PEEP according to inspiratory and/or expiratory transpulmonary pressure) [26]. Respiratory rate (RR) was set to maintain an arterial plasma pH of 7.20 to 7.45 without an increase in intrinsic PEEP.

Extracorporeal membrane oxygenation technics (ECMO) were proposed after at least 12 h of PP for persistent P_a_O_2_/F_i_O_2_ < 60 or respiratory acidosis (pH < 7.2), despite hemodynamic optimization and PEEP adjusted according to esophageal pressure. The ARDS should not last more than 7 days. And the patient also had to be stable enough to support the transfer because our center did not perform ECMO.

In the supine position, the head was elevated between 30° and 45°. In the prone position, the head was elevated between 10° and 20°. Recruitment maneuvres were performed only in case of de-recruiting event (ventilator disconnection, bronchoscopy, prolonged tracheal suctioning, etc.), with a 15-s inspiratory pause at 30 cmH2O.

All patients had an arterial line catheter. They were ventilated with a Hamilton S1 ventilator (Hamilton Medical AG^®^, Bonaduz, Switzerland) equipped with a dedicated proximal pneumotachometer and a dedicated main stream volumetric capnography sensor (Capnostat-5^®^, Hamilton Medical AG^®^, Bonaduz, Switzerland). Pulmonary mechanics, data from volumetric capnography and arterial blood gas were recorded before prone positioning, 2 h after proning, before return in SP and 2 h after return in SP. Static values were recorded and calculated at these steady-state periods. Additionally end-tidal CO_2_ pressure (P_et_CO_2_), dynamic compliance (C_dyn_), VT_e_, physiological Bohr dead space (*V*_D_/*V*_T-phy_) and phase 3 slope of the volumetric capnography (S_III_) were recorded before proning and every 30 min during the PP session (until 24 h of PP). S_III_ of volumetric capnography is a plateau phase corresponding to the emptying of the alveolar compartment. The more flat the slope (i.e., close to 0), the more the alveoli have a simultaneous emptying and an identical CO_2_ concentration (therefore similar ventilation/perfusion ratios). The increase in the slope will thus be associated with a desynchronization of the alveolar emptying and/or an increase in the inhomogeneity of the ventilation/perfusion ratios [27]. Dynamic driving pressure (Δ*P*_dyn_) was calculated by VT_e_/C_dyn_.

Baseline characteristics at the inclusion were recorded: age, gender, body mass index, severity scores (SAPS 2 and SOFA), ARDS (according to Berlin definition) [28] and the etiology of acute respiratory failure. The number of PP sessions and their lengths were recorded. Associated ICU main treatments (length of mechanical ventilation, use (at any time) of non-invasive ventilation, vasopressors, tracheostomy, renal replacement therapy, extracorporeal membrane oxygenation, corticosteroids and NMBA) were detailed as well as patients’ outcomes.

Wilcoxon matched pairs rank tests were performed to compare patients’ characteristics before PP, 2 h after PP, before SP and 2 h after SP. To evaluate time effect, responder sessions were defined as the change between baseline value and end session value (before return in SP), greater than or less than 0% according to the expected physiological effect (e.g., C_dyn_-responder sessions were assessed by Δ*C*_dyn_ > 0%; Δ*P*_dyn_ responder sessions were assessed by ΔΔ*P*_dyn_ < 0%…). Each PP session could be a responder session for one parameter, independently of the response to other parameters. All sessions were analyzed with the parameters collected every 30 min: P_et_CO_2_, C_dyn_, *V*_D_/*V*_T-phy_, S_III_ and Δ*P*_dyn_. For the secondary objective, data from the first PP session were compared between survivors and deaths. Mann–Whitney and Fisher exact tests were used to compare patients’ characteristics according to their vital status on hospital discharge. We also performed multivariate logistic regression analyses with mortality as the dependent variable. We analyzed data from the first PP session and the effect on the main responding physiological parameters for each patient. The regression models were then tested by Hosmer and Lemeshow goodness-of-fit test. Statistical analyses were performed with Prism 6 (GraphPad Software Inc^®^, San Diego, CA, USA) and SPSS Statistics V20 (IBM^®^, New York, NY, USA).

The study protocol has been approved by the Institutional Review Board of the French learned society for respiratory medicine (Société de Pneumologie de Langue Française) in accordance with the ethical standards laid down in the 1964 Declaration of Helsinki and its later amendments. Patients, or their relatives, have consented to the use of the data. The trial has been recorded in ClinicalTrials.gov (NCT 02816190).

## Results

112 patients were eligible for inclusion. 9 were not included because of severe hemodynamic compromise (*n* = 5), missing data at inclusion (*n* = 3; one of which was transferred early for ECMO) and therapeutic limitation decision (*n* = 1). 103 patients were included performing 231 PP sessions (2.2 ± 1.8 PP sessions per patient) with a mean length of 21.5 ± 5 h per PP session. 10 (4.3%) PP sessions were interrupted before 16 h and only 2 (0.8%) before 12 h (one for surgery, one for cardiac arrest). The longest session lasted 41.5 h. Baseline characteristics at inclusion, main ICU treatments, complications during ICU care and outcomes are summarized in Table 1. These were mainly severe patients (SAPS 2 = 54 ± 17) with ARDS (95%) due to pneumonia (93.2%) and with septic shock (98.1%). 5 (5%) patients were prone positioned for acute hypoxemic failure due to unilateral pneumonia. Virtually all patients were paralyzed (94.2%). 38 patients died in ICU (36.9%) and 39 (37.9%) during hospital stay. Complications specifically related to PP have not been systematically collected. However, there were no serious complications: no tearing of catheters or tracheal tubes. One cardiac arrest was noted during the PP, occurring in an already moribund patient before the reversal. Complications were limited to pressure sores and occasional transient airway obstructions occurring just after the PP maneuver.

**Table 1:** Patients’ characteristics at inclusion, during intensive care unit stay and main outcomes

Patients’ variables	Values
Inclusion	
*N*	103
Age (years)	60 ± 13 (58–63)
Male gender (*n*)	73 (70.9%)
BMI (kg/m^2^)	29 ± 7 (28–31)
SAPS2	54 ± 17 (51–57)
SOFA	9 ± 4 (9–10)
ARDS (*n*)	98 (95%)
ARDS due to pneumonia (*n*)	96 (93.2%)
ICU stay	
Prone position sessions (*n*)	231
Prone position sessions (*n* per patient)	2.2 ± 1.8 (1.9–2.6)
Prone position sessions (hours)	21.5 ± 5 (20.7–22)
Delay between sessions (hours)	64.2 ± 99.2 (37.3–91.2)
Invasive mechanical ventilation (days)	17.3 ± 10.9 (15.2–19.4)
Non-invasive ventilation (*n*)^a^	73 (70.9%)
Non-invasive ventilation (days)^a^	3.2 ± 2.6 (2.6–3.8)
Vasopressor (*n*)^a^	101 (98.1%)
Vasopressor duration (days)^a^	9 ± 7.3 (7.6–10.5)
Tracheostomy (*n*)^a^	10 (9.7%)
Renal replacement therapy (*n*)^a^	29 (34%)
Corticosteroids (*n*)^a^	68 (66%)
NMBA (*n*)^a^	97 (94.2%)
Extracorporeal membrane oxygenation (*n*)^a^	1 (1%)
Complications and outcomes	
Pneumothorax (*n*)^a^	2 (1.9%)
Ventilation-associated pneumonia (*n*)^a^	44 (49.7%)
ICU length of stay (days)	22.3 ± 13.8 (19.6–24.9)
Hospital length of stay (days)	27.2 ± 15.5 (24.2–30.1)
ICU deaths with ARDS (*n*)	34 (33%)
ICU deaths (*n*)	38 (36.9%)
Hospital deaths (*n*)	39 (37.9%)

Values are expressed with n (%) or mean ± SD (95% confidence interval)

*BMI* body mass index, *ARDS* acute respiratory distress syndrome, *NMBA* neuro-muscular blocking agents, *ICU* intensive care unit

^a^At any time within ICU stay

Ventilator settings, lung mechanics and arterial blood gas at admission are detailed in Table 2. Mean P_a_O_2_/F_i_O_2_ was 129 ± 52 with total PEEP (PEEP_tot_) 16 ± 3 cmH_2_O and VT_e_ 7 ± 1.5 mL/kg predicted body weight, generating a plateau pressure (P_plat_) of 29 ± 4 cmH_2_O and a driving pressure (Δ*P* = *P*_plat_ − PEEP_tot_) of 13.7 ± 4.7 cmH_2_O. Only 5 (4.9%) patients among 15 (14.6%) equipped, had a change in PEEP, before PP, based on esophageal pressure values. These changes were not related to the patients’ position because the esophageal pressure values were similar in PP and SP.

**Table 2:** Arterial blood gas, lung mechanics and volumetric capnography data during the first PP session

Parameters	Before PP	2 h after PP	Before SP	2 h after SP
pH	7.26 ± 0.1	7.29 ± 0.1^a^	7.35 ± 0.1^a^	7.34 ± 0.1^a^
P_a_O_2_ (mmHg)	77 ± 32	99 ± 60^a^	83 ± 23^a^	79 ± 23
P_a_CO_2_ (mmHg)	54 ± 13	51 ± 15^a^	46 ± 11^a^	46 ± 13^a^
Bicarbonates (mmol/L)	23.9 ± 5.4	23.5 ± 5.6^a^	25 ± 5.9^a^	24.3 ± 5.4
S_a_O_2_ (%)	94 ± 3	96 ± 2^a^	97 ± 2^a^	96 ± 3^a^
RR (cycles/min)	24 ± 6	25 ± 6^a^	26 ± 6^a^	26 ± 6^a^
*V*T_e_ (mL/kg PBW)	7 ± 1.5	7.1 ± 1.9	7.2 ± 1.9	7.1 ± 1.7
F_i_O_2_ (%)	65 ± 22	54 ± 19^a^	39 ± 14^a^	46 ± 17^a^
*P*_plat_ (cmH_2_O)	29 ± 4	28 ± 4	27 ± 3^a^	27 ± 4
PEEPtot (cmH_2_O)	16 ± 3	16 ± 3	15 ± 3	16 ± 3
*C*_stat_ (mL/cmH_2_O)	39 ± 16	40 ± 15	44 ± 18^a^	45 ± 19^a^
P_a_O_2_/F_i_O_2_	129 ± 52	189 ± 79^a^	237 ± 92^a^	192 ± 79a
Δ*P* (cmH_2_O)	12.7 ± 4	12.3 ± 3.9	11.6 ± 3.8^a^	11.6 ± 4.3
S_III_ (%CO_2_/L)	9.28 ± 6.83	8.74 ± 8.5	10.1 ± 20.5	7.05 ± 5.18^a^
P_et_CO_2_ (mmHg)	41 ± 9	39 ± 8^a^	37 ± 8^a^	35 ± 7^a^
VCO_2-min_ (mL/min)	226 ± 71	232 ± 64	227 ± 64	225 ± 62
*V*_D_/*V*_T-phy_ (%)	36.6 ± 8.5	35.9 ± 7.9	34.9 ± 8	33.5–8^a^
*P*_(a-et)_CO_2_/P_a_CO_2_ (%)	22 ± 16.5	20.7 ± 15.3	19.5 ± 15.1^a^	20.4 ± 17.8
*V*_E_/VCO_2-min_	66.5 ± 132	49.2 ± 13	67.8 ± 161^a^	61.2 ± 94.9^a^

Values are expressed with mean ± SD

*RR* respiratory rate, *C*_stat_ static compliance, Δ*P* driving pressure, *S*_III_ phase 3 slope of volumetric capnography, *V*_E_ minute ventilation

^a^*p* < 0.05 compared to baseline value (before PP)

The evolutions of lung mechanics, arterial blood gas and ventilator settings are specified in Table 2 and Table S1, Additional file 1 for the first PP session and in Table S2, Additional file 1 for all PP sessions combined. They globally showed similar results: a significant increase in pH, static compliance (C_stat_) and P_a_O_2_/F_i_O_2_ with a significant decrease in P_a_CO_2_, P_plat_, S_III_, P_et_CO_2_, *V*_D_/*V*_T-phy_ and Δ*P*. There was also an increase in mechanical power, probably secondary to slight modifications of RR and inspiratory/expiratory ratio (parameters included in the formula used) [29]. The decrease in Δ*P* was the only parameter significantly associated with an increase in P_a_O_2_/F_i_O_2_ > 50% (Table S3, Additional file 1).

**Fig. 1:**
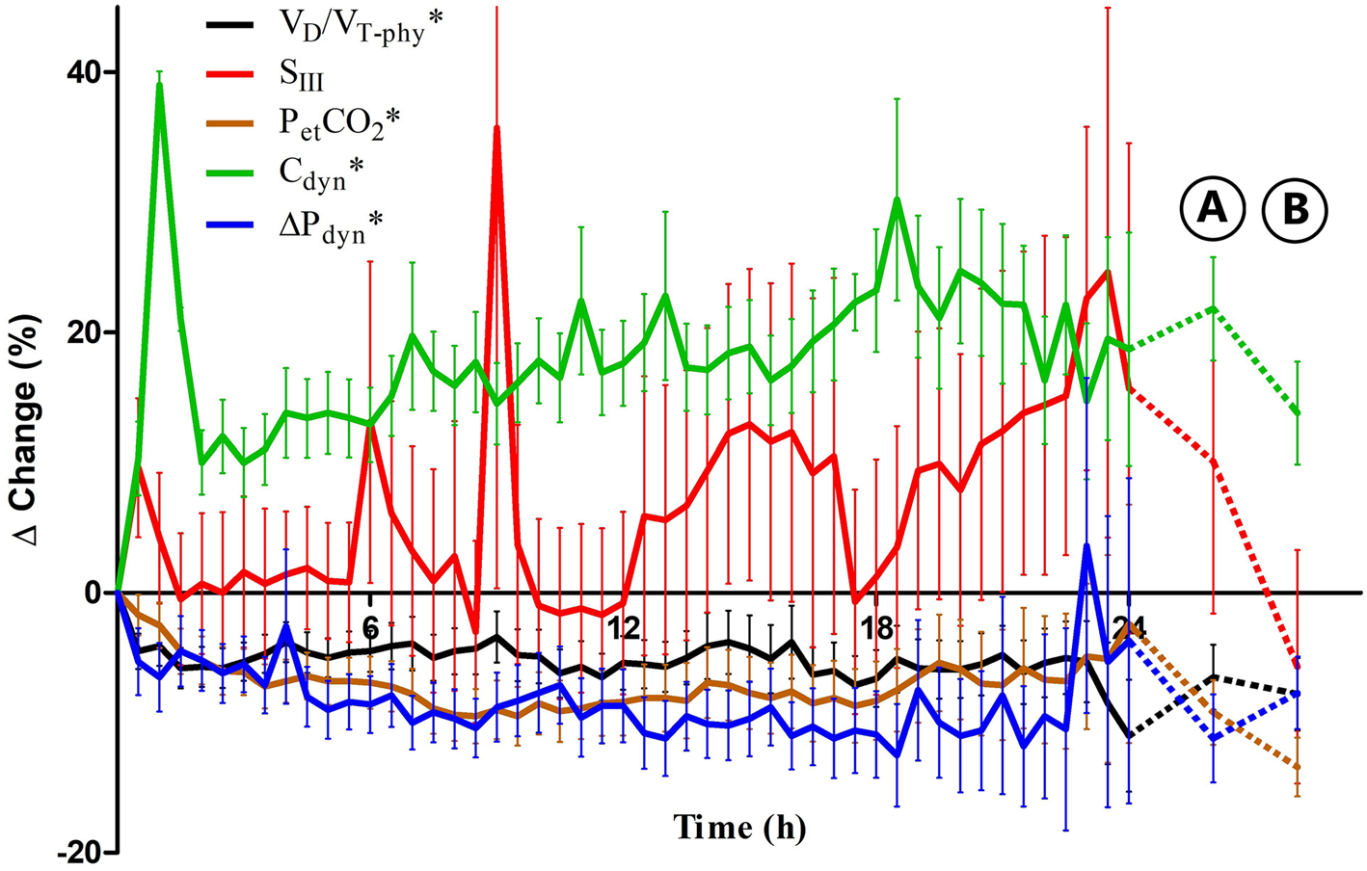
Evolution of Δ*V*_D_/*V*_T-phy_, Δ*S*_III_, Δ*P*_et_CO_2_ and Δ*C*_dyn_ for each parameter (session 1), from 0 h (just before prone positioning) to 24 h of prone position; Ⓐ at sessions’ end (dotted line); Ⓑ 2 h after return in supine position (dotted line). *p < 0.05 (global time effect comparison)

**Fig. 2:**
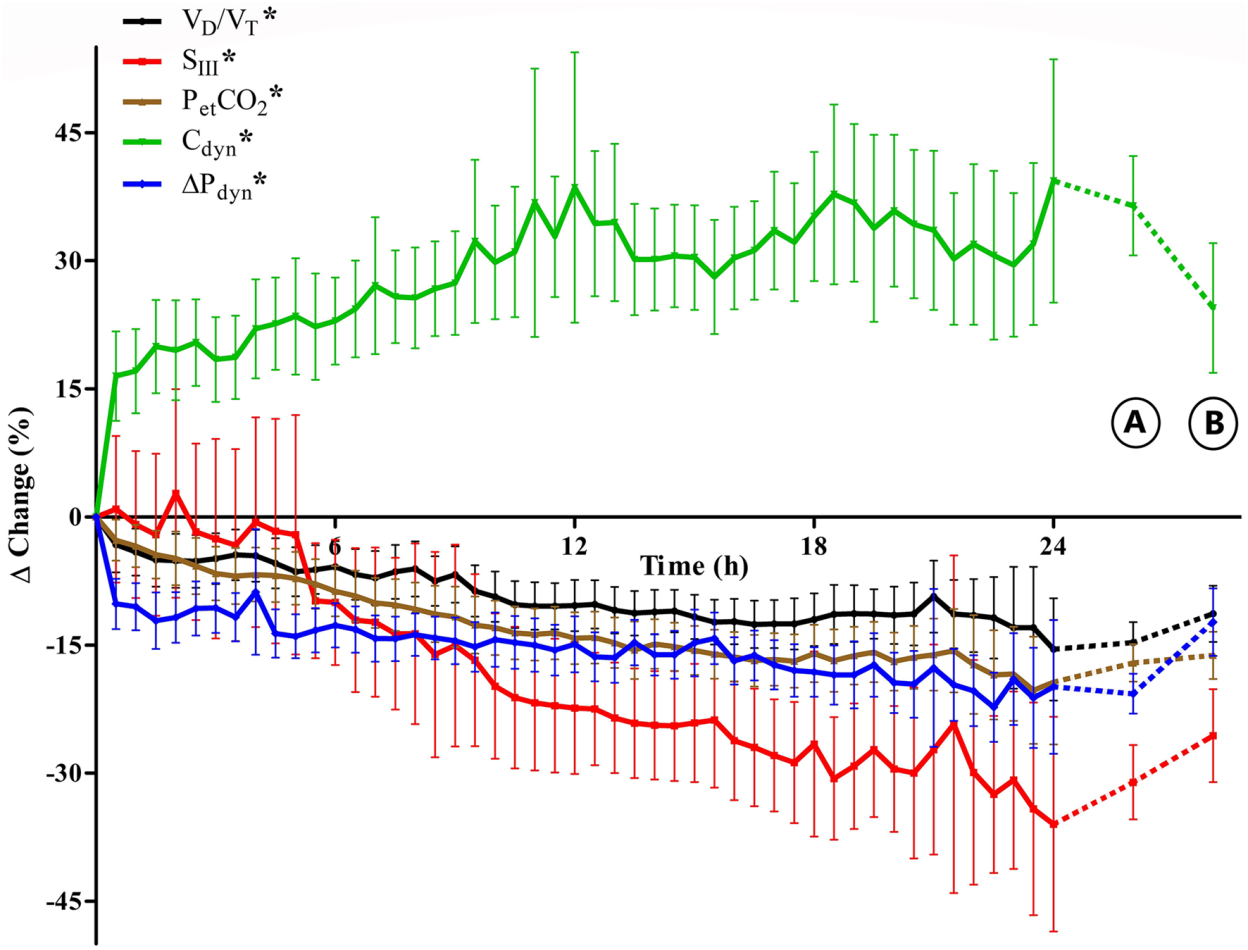
Evolution of Δ*V*_D_/*V*_T-phy_, ΔS_III_, Δ*P*_et_CO_2_ and Δ*C*_dyn_ in responder sessions for each parameter (all sessions), from 0 h (just before prone positioning) to 24 h of prone position; Ⓐ at sessions’ end (dotted line); Ⓑ 2 h after return in supine position (dotted line). *p < 0.05 (global time effect comparison)

We pooled data evolutions from the baseline (before PP) of the first session (Fig. 1) and of all PP sessions (Figure S1, Additional file 1), sampled every 30 min. We could observe a rapid increase not only in C_dyn_, but also in S_III_. Δ*P*_dyn_ and P_et_CO_2_ decreased slightly. There was a significant decrease in *V*_D_/*V*_T-phy_ during the first session, but not during the pooled sessions. The pooled responder sessions to *V*_D_/*V*_T-phy_ (number of responder sessions = 77 (46.7%)), S_III_ (87 (52.4%)), P_et_CO_2_ (118 (61.8%)), C_dyn_ (131 (73.2%)) and Δ*P*_dyn_ (136 (76%)) showed that the beneficial physiological effect continued after 16 h of PP and at least up to 24 h which was the maximum limit for recording data (Fig. 2). When we have only looked at the first PP sessions, the response session rate was similar to that of pooled sessions: *V*_D_/*V*_T-phy_ 44 (61.1%), S_III_ 47 (65.3%), P_et_CO_2_ 65 (69.9%), C_dyn_ 62 (76.5%) and Δ*P*_dyn_ 65 (80.2%). There were 90 (91.8%) P_a_O_2_/F_i_O_2_ responders.

The duration of PP allowing to obtain the maximum effect was different for each parameter studied and was in median between 16 h [13, 20] and 18 [14, 21] for all sessions, between 16 h [14, 19] and 19 [16, 23] for responder sessions and between 17 h [13, 22] and 18 [14, 21] for session 1 (Table S4, Additional file 1). The parameter with the higher beneficial variation was C_dyn_ with a mean 35% increase in value for the first PP session up to 40% in C_dyn_-responder sessions (Figs. 1, 2). Non-responder sessions are shown in Additional file 1: Figure S2.

The search of predictive criteria for mortality before prone positioning (Table S5, Additional file 1) found that higher age (67.6 ± 12.6 years versus 56.7 ± 12.1; *p* < 0.001), higher SAPS 2 (63 ± 17 vs 49 ± 15, *p* < 0.001), lower C_stat_ (33 ± 14 vs 43 ± 16; *p* = 0.003) and a lower minute CO_2_ production (VCO_2-min_) (185 ± 81 mL/min vs 250 ± 53; *p* < 0.001) were associated with mortality, whereas P_a_O_2_/F_i_O_2_ (*p* = 0.659), Δ*P* (*p* = 0.096) and *V*_D_/*V*_T-phy_ (*p* = 0.37) were not.

The evolution of the respiratory parameters during the first session, compared (without adjustment) between the survivors and the dead, did not clearly find any specific response to PP, whether during the session or 2 h after the return in SP (Tables S6, S7 and S8, Additional file 1). In multivariate analysis (before PP-before SP), the increase in VCO_2-min_ (maybe mediated by the hemodynamic effects of PP) and in *V*_D_/*V*_T-phy_ were associated with mortality (respectively, odds ratio 1.04 (95% confidence interval 1.01; 1.07; *p* = 0.015) and 1.03 (1; 1.05; *p* = 0.031)) (Table S6, Additional file 1). In the first session (before PP-after SP), there was also a significantly greater drop in dead space among survivors (Δ*V*_D_/*V*_T-phy_ = − 9% [− 20; 0.27] vs − 0.59% [− 11.2; 9.87]; *p* = 0.036). However, this effect was not found again in multivariate analysis (Table S7, Additional file 1).

It is generally observed that the parameters improving during PP as P_a_O_2_/F_i_O_2_ are degraded after return in SP. It could be estimated that the intensity of this relapse after return in SP is associated with excess severity or mortality of patients. However, there was no significant association between the intensity of this relapse and the mortality of patients in the limit of potential biases (Tables S9, Additional file 1).

**Fig. 3:**
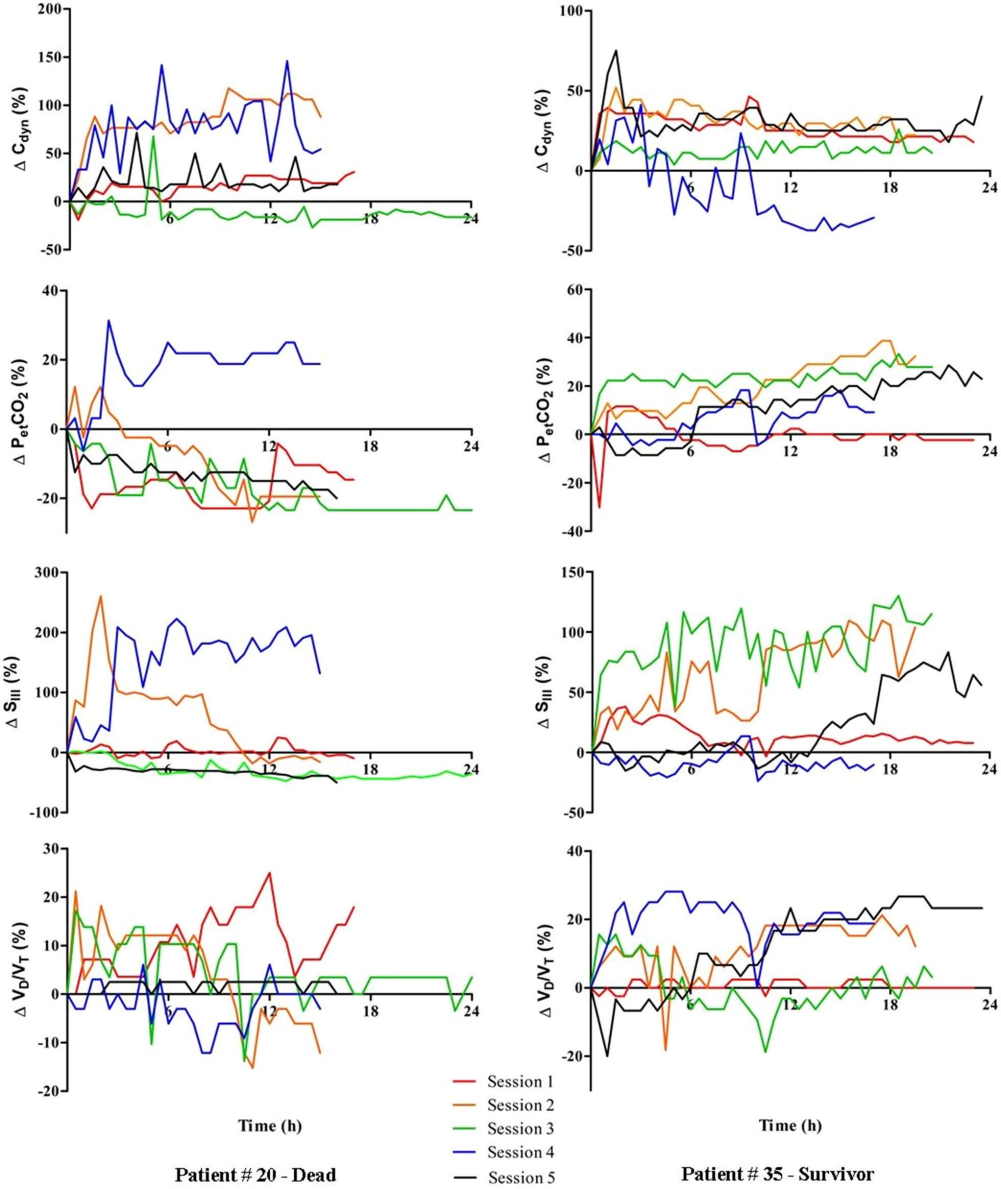
Evolution over five consecutive prone position sessions of Δ*C*_dyn_, Δ*P*_et_CO_2_, ΔS_III_ and Δ*V*_D_/*V*_T-phy_, in patient #20 (dead) and patient #35 (survivor), from 0 h (just before prone positioning) to the end of each prone position session

We have also noticed a wide variability of response to the positioning of the respiratory parameters between each session and in the same patient. Figure 3 shows the evolution over five consecutive PP sessions of Δ*V*_D_/*V*_T-phy_, ΔS_III_, Δ*P*_et_CO_2_ and Δ*C*_dyn_ in patient #20 (dead) and patient #35 (survivor). The responder or non-responder character for a parameter varied according to the patient but also according to the session and the time of the session (after 6 h, 12 h, 20 h…). In addition, for the same session, a patient could respond to one parameter but not to another. And it could be the opposite for the next session.

## Discussion

Our study shows that the physiologic effects of PP increase for at least 24 h in some patients. It also shows that it is impossible to predict which patient will benefit from this technique before its introduction, during or even after the return in SP. The analysis of the respiratory parameters before, during and after the sessions, as well as the variability of the effect of repeated sessions in the same patient, renders the concept of PP responder lacking in physiological basis.

It has already been shown that the oxygenation gain estimated by the P_a_O_2_/F_i_O_2_ was not associated with a reduction in mortality thanks to PP [30]. It is the same for all the other parameters studied here, although previous studies have found potentially prognostic parameters such as the decrease in P_a_CO_2_ [31, 32] or alveolar dead space [32]. These results are in fact logical if one relies on the mechanism of action of prone positioning, which consists of a reduction of the VILI. PP reduces lung stress and strain by reducing the overdistention of anterior non-dependent zones while allowing recruitment of posterior dependent zones [7–9, 13, 14]. Lung expansion is more constant and homogeneous [10, 15, 16]. It should therefore be considered that putting on PP is an integral part of protective ventilation techniques such as reducing the VT. In the same way that the use of reduced current volumes should be applied until a significant improvement in ARDS allowing the reintroduction of spontaneous ventilation, PP sessions should probably be extended to a similar time. It has, again recently, been shown that the recruitment of posterior lung zones depends on the duration of positioning [33]. In our study the beneficial effect continued to increase after at least 24 h. Interestingly, the PROSEVA study [6], the only positive randomized controlled trial, was the only one that did not stop PP therapy after a pre-established period, but continued until a significant (P_a_O_2_/F_i_O_2_ > 150 in SP) and persistent (> 4 h) improvement occurred. It would seem reasonable to continually leave patients in PP until the overall improvement of their condition allows sedation to be stopped and spontaneous ventilation to resume. Rather than repeating the sessions, it would probably be better to extend the sessions. The beneficial effect is linked to the length spent in PP, not to the maneuver per se. Also, complications during turning maneuvers are rare in experienced teams, but probably more frequent in the smallest centers. If our study did not allow us to predict a benefit in extending the sessions for more than 24 h, it nevertheless invited us to make them last at least 24 h. It also does not seem logical to interrupt sessions, whose initiation was motivated by a P_a_O_2_/F_i_O_2_ of less than 150, if this P_a_O_2_/F_i_O_2_ remains below 150 after 24 h. Therefore, we suggest that the PP sessions last at least 24 h and be extended in the event that the P_a_O_2_/F_i_O_2_ ratio at 24 h remains below 150.

Preventing ventilation-induced injuries is crucial for the future of patients with ARDS. Most of these patients die, while still on mechanical ventilation, but rarely with refractory hypoxemia [34]. It is mainly the overcomplications, the sequelae of the causal disease and the maintenance of pulmonary lesions by the mechanical ventilation which prolong the duration of invasive ventilation and are responsible for the mortality. PP should not be used as a rescue, but as a routine therapy in ARDS patients. Yet a multicenter study in 2017 found that the prevalent reason for not using PP was that hypoxemia was not considered sufficiently severe [35]. Future studies should focus on researching the benefit of PP in all ARDS patients regardless of the severity stage. There were no life-threatening complications in our cohort and they are described as rare [6, 35]. The benefit/risk balance is therefore strongly in favor of the PP in moderate-to-severe ARDS. It should be evaluated in the mild-to-moderate ARDS. Of course, the PP session could be interrupted in the event of a serious complication or urgent care.

This study presents several limitations. First, the duration of the sessions was left free with an obligation of minimum duration of 16 h. These lasted 21.5 ± 5 h (20.7–22) and there was no difference in duration between surviving patients and deaths (respectively, 20.8 ± 3 h vs 21.1 ± 6.5; *p* = 0.719) (Tables S1, S2 and S3, Additional file 1). In the same way, some modifications of the ventilators’ settings were allowed (RR and PEEP). This could partially explain the improvement in some studied parameters. As Tables S1, S2 and S3, Additional file 1 show, these changes may be statistically significant but are not clinically relevant. They cannot explain the observed evolution of physiological parameters by themselves. The design of the study does not allow, without a control group, to affirm that the physiological effects studied are solely due to PP and not to the evolution of the disease. Especially since part of the effect is “lost” after the return to SP. This effect is really related to PP for P_a_O_2_/F_i_O_2_ [6], for pH and RR (therefore for P_a_CO_2_) [4] and therefore also for the other studied parameters that evolved very significantly over short periods.

The absence of randomization meant that the analysis of the factors potentially associated with mortality was marred by many potential biases. It was an observational study and these results should be interpreted with caution.

Another major limitation concerns the population studied. These were mainly patients with ARDS secondary to pneumonia complicated by septic shock. It is possible to say that the results could have been different with extrapulmonary ARDS [36] or with less shocked patients. However, this would not change the logic of leaving patients in PP to reduce the risk of VILI.

Anatomical or functional alveolar recruitability has not been evaluated. It would be an interesting parameter to evaluate in the effects of PP. Finally, the hemodynamic impact of PP has not been studied except via VCO_2-min_ and P_(a-et)_CO_2_. Indeed our study found an association between mortality and low CO_2_ production. It was probably linked to a defect in cellular perfusion from circulatory failure. 101 (98.1%) of the patients were on vasopressors. This aspect is probably major, at least in patients with acute cor pulmonale [37, 38]. More studies are needed on this topic. In addition, the volumetric capnography data are significantly affected by the hemodynamic state of the patient, which makes their interpretation more difficult.

## Conclusions

In conclusion, the maximum physiological response to PP requires in some patients at least 24 h of positioning. It is not possible to predict which patient will benefit from PP before, during, or even after the maneuver. Therefore, we suggest that the PP sessions last at least 24 h and be extended in the event that the P_a_O_2_/F_i_O_2_ ratio at 24 h remains below 150. In the event of a relapse of the P_a_O_2_/F_i_O_2_ ratio below 150 after putting back in supine position, a new session of at least 24 h of PP should be carried out, and so on until the moment of deciding to stop sedation and resume spontaneous ventilation. PP should be integrated into the protective ventilation of moderate-to-severe ARDS in the same way as the reduction in tidal volume.

## Supplementary information

**Additional file 1.** Additional figures and tables.

## Data Availability

Deidentified patients’ datasets used and/or analyzed during the current study are available from the corresponding author on reasonable request.

## Abbreviations

ARDS: Acute respiratory distress syndrome
BMI: Body mass index
C_dyn_: Dynamic compliance
C_stat_: Static compliance
Δ*P*: Driving pressure
Δ*P*_dyn_: Dynamic driving pressure
ECMO: Extracorporeal membrane oxygenation
ICU: Intensive care unit
NMBA: Neuromuscular blocking agent
P_(a-et)_CO_2_: Difference between arterial and end-tidal CO_2_ pressures
PBW: Predicted body weight
PEEP_tot_: Total PEEP
P_et_CO_2_: End tidal CO_2_ pressure
PP: Prone position
P_plat_: Plateau pressure
RR: Respiratory rate
S_III_: Phase 3 slope of volumetric capnography
SAPS 2: Simplified Acute Physiologic Score 2
SOFA: Sepsis-related Organ Failure Assessment
SP: Supine position
VCO_2-min_: CO_2_ production per minute
*V*_D_/*V*_T-phy_: Physiological Bohr dead space
V_E_: Minute ventilation
VILI: Ventilator-induced lung injury
VT: Tidal volume
VT_e_: Expiratory tidal volume
