# Supplementary material for: Duration of prone position sessions: a prospective cohort study
Source: Ann Intensive Care. 2020 May 24;10:66. doi: 10.1186/s13613-020-00683-7 (PMC7245995; doi:10.1186/s13613-020-00683-7)
Supplement: Supplementary file 1 — Additional file 1. Additional figures and tables. [file 13613_2020_683_MOESM1_ESM.doc]

**ADDITIONAL FILE 1 :**

**Additional DIGITAL CONTENT**

**Original Title: Duration of prone position sessions: a prospective cohort study**

Authors: Sebastien JOCHMANS, MD (1,2) *, Sandie MAZERAND, MD (1), Jonathan CHELLY, MD (1,2), Franck POURCINE, MD (1), Oumar SY, MD (1), Nathalie THIEULOT-ROLIN, MD (1), Olivier ELLRODT, MD (1), Emmanuelle MERCIER DES ROCHETTES, MD (1,3), Gaël MICHAUD (1), Jean SERBOURCE-GOGUEL, MD (1), Christophe VINSONNEAU, MD (1,2,4), Ly Van Phach VONG, MD (1), Mehran MONCHI, MD (1,2)

*Corresponding Author : Sebastien JOCHMANS. Département de Médecine Intensive-Réanimation, GH Sud Ile-de-France, Hôpital de Melun, 270 avenue Marc Jacquet, 77000 Melun, France. [sebastien.jochmans@gmail.com](mailto:sebastien.jochmans@gmail.com). Phone: 33-181742078. Fax: 33-181742205

**ADDITIONAL FILE CONTENT:**

- **Abbreviations**
- **Prone Positioning Protocol**
- **Table S1.** Arterial blood gas, lung mechanics and volumetric capnography data during the first prone position session.
- **Table S2.** Arterial blood gas, lung mechanics and volumetric capnography data during all Prone Position sessions.
- **Table S3.** Factors associated with a marked increase in P/F ratio. Univariate and multivariate analysis.
- **Table S4.** Time to maximum effect between 0 to 24 hours (limit of record).
- **Table S5.** Univariate analysis of patients’ characteristics at inclusion during ICU and hospital stays between dead and survivors.
- **Table S6.** Multivariate analysis of Δchange, before PP-before SP, in respiratory parameters between dead and survivors during the first PP session.
- **Table S7.** Multivariate analysis of Δchange, before PP-2 hours after SP, in respiratory parameters between dead and survivors during the first PP session.
- **Table S8.** Multivariate analysis of responding parameters during PP session and after PP session, regarding ICU death.
- **Table S9.** Univariate analysis of Δchange, before SP-2 hours after SP, in respiratory parameters between dead and survivors during the first PP session.
- **Table S10.** Lung mechanics and outcome regarding first and average PP sessions.*
- **Table S11.** Arterial blood gas, lung mechanics and volumetric capnography data during average Prone Position sessions per patient.*
- **Table S12.** Multivariate analysis of Δchange, before PP-before SP, in respiratory parameters between dead and survivors during the average PP session per patient.*
- **Table S13.** Multivariate analysis of Δchange, before PP-2 hours after SP, in respiratory parameters between dead and survivors during the average PP session per patient.*
- **Table S14.** Univariate analysis of Δchange, before SP-2 hours after SP, in respiratory parameters between dead and survivors during the average PP session per patient.*
- **Figure S1.** Evolution of ΔV_D_/V_T-phy_, ΔS_III_, ΔP_et_CO_2_ and ΔC_dyn_ for each parameter (all sessions), from 0 hour (just before prone positioning) to 24 hours of prone position; at sessions’ end; 2 hours after return in supine position.
- **Figure S2.** Evolution of ΔV_D_/V_T-phy_, ΔS_III_, ΔP_et_CO_2_ and ΔC_dyn_ for each parameter (all non-responders sessions), from 0 hour (just before prone positioning) to 24 hours of prone position; at sessions’ end; 2 hours after return in supine position.

* For each patient, the effects of the different PP sessions were also averaged to obtain an “average PP session” per patient. The objective of this averaging was to reduce the intra-individual variability that could be linked to an external event (unstable hemodynamics, atelectasis, bronchial endoscopy…).

**Abbreviations**

ARDS : Acute Respiratory Distress Syndrome

BMI : Body Mass Index

C_dyn_ : Dynamic Compliance

C_stat_ : Static Compliance

DP : Driving Pressure

DP_dyn_ : Dynamic Driving Pressure

ECMO : Extracorporeal Membrane Oxygenation

F_e_CO_2_ : Expired Fraction of CO_2_

ICU : Intensive Care Unit

NMBA : Neuromuscular Blocking Agent

P_(a-et)_CO_2_ : Difference between Arterial and End-Tidal CO_2_ pressures

PBW : Predicted Body Weight

PEEP_tot_ : Total PEEP

P_et_CO_2_ : End Tidal CO2 Pressure

PP : Prone Position

P_plat_ : Plateau Pressure

P_max_ : Maximum Airway Pressure

R_aw_ : Airways Resistance

RR : Respiratory Rate

S_III_ : Phase 3 Slope of Volumetric Capnography

S_III-R_ : Ratio Observed/Theoretical Phase 3 Slope of Volumetric Capnography

SAPS 2 : Simplified Acute Physiologic Score 2

SOFA : Sepsis-related Organ Failure Assessment

SP : Supine Position

VCO_2-min_ : CO2 production per minute

Vds : Dead Space Volume per breath

V_D_/V_T-phy_ : Physiological Bohr Dead Space

V_E_ : Minute Ventilation

VILI : Ventilator Induced Lung Injury

VT : Tidal Volume

VT_e_ : Expiratory Tidal Volume

**Prone Positioning Protocol**

**Equipment preparation**

- 2 bolsters (thorax and legs), scope electrodes, sheets, hard mattress, skin protection pads

**Patient’s preparation**

- Taking hemodynamic and respiratory constants
- Arterial blood gases
- Hydration and occlusion of the eyes, mouth care
- Verification of the position (noted mark) and fixation of the tracheal tube
- Check pipe attachments and anticipate their length
- Skin protection pads without crease on the points of support (chin, iliac spines, knees, tibial ridges and foot-stumps) and the injured areas
- Setting in FiO_2_ 100%
- Endotracheal aspiration

**Maneuver**

- Decide on the side of the upturn (depending on the length of the pipes and preferably opposite the catheters)
- 4 to 5 (if obese patient) people including a doctor. Doctor at the head, 2 on the turning side, 1 to 2 on the other side)
- Role of the doctor: directs and supervises the gesture; lead, watch the intubation tube and the nasogastric tube ; head restraint during rotation; monitors scope and ventilator
- Transfer to the edge of the bed with the sheet then turn on the side ("slice"), the arm along the body
- Remove the patches from the scope and put them on the back, ensure the length of the pipes
- Lay a sheet without fold on the bed, an absorbent pad that will be under the head and another under the perineum
- Tilt the patient to return and refocus
- Pull the sheet by removing the folds and foreign bodies under the patient
- Put a bolster under the thorax at the level of the shoulders (between mattress and sheet) so that the face does not crash on the mattress and put a bolster under the shins / kicks to avoid the position in equine of the feet
- Tilt the bed at 10 °
- Check the reference of the intubation probe (reposition if necessary) as well as the pipe fixings and their permeability
- Endotracheal aspiration and taking hemodynamic and respiratory measures. Note prone position hour in the ICU software

**Oversight**

- Endotracheal aspiration usually frequent at the beginning of the session
- Blood gas 2h after prone positioning
- Surveillance and standard care, no crawl

**Length**

- 16 hours minimum

**Nutrition**

- Metoclopramide and Erythromycin Systematic at Least for the First PP Session

**Return in Supine Position**

- Same maneuver, same precautions, same supervision

**Tables**

**TABLE S1. Arterial blood gas, lung mechanics and volumetric capnography data during the first prone position session.**

| **Paramètres** | **Before PP** | **2 hours after PP** | **Before SP** | **2 hours after SP** |
| --- | --- | --- | --- | --- |
| **pH** | 7.26±0.1 (7.24-7.28) | 7.29±0.1 (7.27-7.31)* | 7.35±0.1 (7.33-7.37)* | 7.34±0.1 (7.33-7.36)* |
| **PaO_2_ (mmHg)** | 77±32 (71-83) | 99±60 (87-111)* | 83±23 (79-88)* | 79±23 (74-83) |
| **PaCO_2_ (mmHg)** | 54±13 (52-57) | 51±15 (48-53)* | 46±11 (44-49)* | 46±13 (44-49)* |
| **Bicarbonates (mmol/L)** | 23.9±5.4 (22.9-25) | 23.5±5.6 (22.4-24.6)* | 25±5.9 (23.8-26.1)* | 24.3±5.4 (23.2-25.4) |
| **SaO_2_ (%)** | 94±3 (93-95) | 96±2 (96-97)* | 97±2 (96-97)* | 96±3 (95-96)* |
| **FiO_2_ (%)** | 65±22 (61-69) | 54±19 (50-58)* | 39±14 (36-41)* | 46±17 (43-49)* |
| **P_moy_ (cmH_2_O)** | 21±3 (21-22) | 21±3 (21-22) | 20±3 (20-21)* | 21±3 (20-21)* |
| **P_plat_ (cmH_2_O)** | 29±4 (28-29) | 28±4 (28-29) | 27±3 (26-28)* | 27±4 (26-28) |
| **PEEP_tot_ (cmH_2_O)** | 16±3 (15-17) | 16±3 (16-17) | 15±3 (15-16) | 16±3 (15-16) |
| **RR (n/min)** | 24±6 (23-26) | 25±6 (24-27)* | 26±6 (24-27)* | 26±6 (24-27)* |
| **VT (mL/kg IBW)** | 7±1.5 (6.7-7.2) | 7.1±1.9 (6.7-7.5) | 7.2±1.9 (6.8-7.6) | 7.1±1.7 (6.7-7.4) |
| **C_stat_ (mL/cmH_2_O)** | 39±16 (36-43) | 40±15 (37-44) | 44±18 (40-48)* | 45±19 (40-49)* |
| **C_dyn_ (mL/cmH_2_O)** | 37±17 (33-41) | 38±15 (35-41)* | 43±18 (39-47)* | 40±16 (36-43)* |
| **ΔP_dyn_ (cmH_2_O)** | 13.7±4.7 (12.7-14.8) | 12.9±3.8 (12.1-13.8)* | 12±4.2 (11.1-12.9)* | 12.3±3.8 (11.5-13.1)* |
| **R_aw_ (cmH_2_O/L/s)** | 14±5 (13-15) | 14±6 (13-16) | 14±7 (13-16) | 14±5 (12-15) |
| **Mechanical Power (J/min)** | 29.3±9.6 (27.3-31.4) | 31.8±10.4 (29.6-34)* | 32.3±11.5 (29.8-34.7)* | 31.1±10.8 (28.8-33.5) |
| **P_max_ (cmH_2_O)** | 36±6 (35-38) | 36±7 (35-38) | 35±7 (34-37)* | 36±6 (35-37) |
| **Vds (mL)** | 159±47 (150-169) | 161±49 (151-171) | 157±40 (148-165) | 151±45 (142-160)* |
| **S_III_ (%CO_2_/L)** | 9.28±6.83 (7.87-10.68) | 8.74±8.5 (7-10.5) | 10.1±20.5 (5.9-14.3) | 7.05±5.18 (5.98-8.12)* |
| **PetCO_2_ (mmHg)** | 41±9 (39-43) | 39±8 (37-40)* | 37±8 (35-38)* | 35±7 (34-36)* |
| **FeCO_2_ (%)** | 5.75±1.31 (5.48-6.02) | 5.48±1.17 (5.24-5.72)* | 5.17±1.09 (4.94-5.39)* | 5±1 (4.8-5.21)* |
| **VCO_2-min_ (mL/min)** | 226±71 (211-241) | 232±64 (219-245) | 227±64 (214-240) | 225±62 (212-238) |
| **PaO_2_/FiO_2_** | 129±52 (118-139) | 189±79 (174-205)* | 237±92 (219-255)* | 192±79 (177-208)* |
| **ΔP (cmH_2_O)** | 12.7±4 (11.8-13.5) | 12.3±3.9 (11.4-13.2) | 11.6±3.8 (10.7-12.5)* | 11.6±4.3 (10.5-12.6) |
| **Oxygenation Index** | 18.8±7.1 (17.4-20.2) | 13.1±5.5 (12-14.2)* | 9.9±4.4 (9.1-10.8)* | 12.9±7 (11.5-14.3)* |
| **S_III-R_** | 3.05±1.92 (2.65-3.44) | 2.91±1.66 (2.58-3.25) | 3.79±9.51 (1.83-5.74) | 2.48±1.44 (2.18-2.77)* |
| **Oxygen Stretch Index** | 11.9±6.3 (10.5-13.3) | 8±5.2 (6.8-9.2)* | 5.9±3.7 (5-6.8)* | 7.1±4.6 (6-8.2)* |
| **V_D_/V_T-phy_ (%)** | 36.6±8.5 (34.8-38.3) | 35.9±7.9 (34.3-37.5) | 34.9±8 (33.3-36.6) | 33.5-8 (31.8-35.1)* |
| **P_(a-et)_CO_2_/PaCO_2_ (%)** | 22±16.5 (18.7-25.3) | 20.7±15.3 (17.6-23.8) | 19.5±15.1 (16.5-22.5)* | 20.4±17.8 (16.8-23.9) |
| **V_E_/VCO_2-min_** | 66.5±132 (39.4-93.6) | 49.2±13 (46.5-51.8) | 67.8±161 (34.7-101)* | 61.2±94.9 (41.5-80.9)* |

Values are mean ± SD (95% Confidence Interval). * p < 0.05 compared to baseline value.

**TABLE S2. Arterial blood gas, lung mechanics and volumetric capnography data during a**verage Prone Position sessions per patient.

| **Parameters** | **Before PP** | **2 hours after PP** | **Before SP** | **2 hours after SP** |
| --- | --- | --- | --- | --- |
| **pH** | 7.29±0.1 (7.27-7.31) | 7.31±0.1 (7.29-7.33)* | 7.36±0.1 (7.35-7.38)* | 7.36±0.1 (7.34-7.37)* |
| **PaO_2_ (mmHg)** | 77±34 (71-84) | 96±54 (86-106)* | 82±21 (78-86)* | 81±35 (74-88) |
| **PaCO_2_ (mmHg)** | 53±12 (51-55) | 50±13 (48-53)* | 48±11 (46-50)* | 47±12 (45-50)* |
| **Bicarbonates (mmol/L)** | 25.1±4.7 (24.2-26) | 24.7±5 (23.8-25.7) | 25.9±5.3 (24.9-27)* | 25.9±7.2 (24.5-27.3) |
| **SaO_2_ (%)** | 94±3 (94-95) | 96±3 (96-97)* | 97±2 (96-97)* | 96±3 (95-96)* |
| **FiO_2_ (%)** | 62±19 (59-66) | 53±17 (49-56)* | 39±12 (37-41)* | 47±17 (43-50)* |
| **P_moy_ (cmH_2_O)** | 21±3 (20-22) | 21±3 (20-21) | 21±8 (20-23) | 21±3 (20-21) |
| **P_plat_ (cmH_2_O)** | 28±4 (28-29) | 28±4 (27-29) | 27±3 (26-28)* | 28±4 (27-29)* |
| **PEEP_tot_ (cmH_2_O)** | 16±3 (15-16) | 16±3 (15-16) | 15±3 (15-16) | 16±3 (15-16) |
| **RR (n/min)** | 25±6 (24-26) | 25±6 (24-26) | 26±6 (25-27) | 26±6 (25-27) |
| **VT (mL/kg IBW)** | 7±1.5 (6.7-7.2) | 7.1±1.8 (6.7-7.4) | 7.6±4.4 (6.8-8.5)* | 7.2±1.6 (6.9-7.5)* |
| **C_stat_ (mL/cmH_2_O)** | 39±16 (36-43) | 41±16 (38-45)* | 44±16 (40-47)* | 44±18 (40-48)* |
| **C_dyn_ (mL/cmH_2_O)** | 38±17 (34-41) | 39±14 (36-42)* | 44±19 (40-48)* | 41±18 (38-45)* |
| **ΔP_dyn_ (cmH_2_O)** | 13.6±4.7 (12.6-14.6) | 12.6±3.7 (11.8-13.4)* | 11.9±4.2 (11-12.8)* | 12.4±4.1 (11.5-13.3)* |
| **R_aw_ (cmH_2_O/L/s)** | 14±5 (13-15) | 15±7 (14-16) | 14±6 (13-15) | 14±6 (13-15) |
| **Mechanical Power (J/min)** | 28.8±9.2 (27-30.6) | 30.2±9.7 (28.3-32.2)* | 31.2±10.8 (29-33.4)* | 30.6±10.1 (28.6-32.6)* |
| **P_max_ (cmH_2_O)** | 36±6 (35-37) | 36±6 (35-37) | 35±6 (34-37)* | 36±6 (35-37) |
| **Vds (mL)** | 154±42 (146-162) | 156±45 (147-164) | 155±38 (148-163) | 151±42 (143-160) |
| **S_III_ (%CO_2_/L)** | 8.17±4.97 (7.19-9.15) | 8.11±4.79 (7.17-9.06) | 9.9±20 (5.86-14) | 7.04±4.95 (6.03-8.04)* |
| **PetCO_2_ (mmHg)** | 40±8 (38-41) | 39±7 (38-40) | 37±7 (36-38)* | 35±7 (34-37)* |
| **FeCO_2_ (%)** | 5.54±1.08 (5.32-5.75) | 5.42±0.94 (5.24-5.61) | 5.21±0.99 (5.01-5.41)* | 5.07±0.94 (4.88-5.26)* |
| **VCO_2-min_ (mL/min)** | 232±63 (219-244) | 233±62 (221-245) | 232±57 (220-243) | 231±54 (220-242) |
| **PaO_2_/FiO_2_** | 134±51 (124-144) | 190±75 (176-205)* | 232±87 (215-249)* | 193±76 (178-208)* |
| **ΔP (cmH_2_O)** | 12.8±4 (11.9-13.6) | 12.2±4 (11.3-13)* | 11.6±3.6 (10.8-12.4)* | 11.8±4.4 (10.8-12.7)* |
| **Oxygenation Index** | 18±6 (16.8-19.1) | 12.9±5 (11.9-13.9)* | 10.7±5.9 (9.5-11.8)* | 13.1±6.9 (11.8-14.5)* |
| **S_III-R_** | 2.72±1.32 (2.46-2.98) | 2.78±1.18 (2.54-3) | 3.73±9.33 (1.85-5.62) | 2.48±1.3 (2.22-2.75)* |
| **Oxygen Stretch Index** | 11.6±6 (10.3-12.9) | 7.9±4.9 (6.9-9)* | 6.3±4.1 (5.4-7.2)* | 7.8±6.1 (6.4-9.1)* |
| **V_D_/V_T-phy_ (%)** | 35.3±7.6 (33.8-36.8) | 34.7±7.1 (33.3-36.1) | 34.5±7.3 (33.1-36) | 33.3±7.2 (31.8-34.8)* |
| **P_(a-et)_CO_2_/PaCO_2_ (%)** | 22.8± 14.7 (19.9-25.6) | 20.7±14.5 (17.9-23.5)* | 20.5±15.6 (17.4-23.6)* | 21.5±17.6 (18-24.9) |
| **V_E_/VCO_2-min_** | 49±15.1 (46-52) | 49.7±13.4 (47.1-52.3) | 51.4±12.2 (48.9-53.9)* | 51.7±12.8 (49.1-54.3)* |

Values are mean ± SD (95% Confidence Interval). * p < 0.05 compared to baseline value.

**TABLE S3. Arterial blood gas, lung mechanics and volumetric capnography data during a**ll Prone Position sessions.

| **Parameters** | **Before PP** | **2 hours after PP** | **Before SP** | **2 hours after SP** |
| --- | --- | --- | --- | --- |
| **pH** | 7.3±0.1 (7.29-7.32) | 7.32±0.1 (7.3-7.33)* | 7.37±0.1 (7.36-7.38)* | 7.36±0.1 (7.34-7.37)* |
| **PaO_2_ (mmHg)** | 74±33 (69-78) | 93±50 (86-100)* | 81±20 (78-84)* | 79±34 (74-83)* |
| **PaCO_2_ (mmHg)** | 52±13 (51-54) | 51±16 (49-53)* | 47±13 (46-49)* | 47±12 (46-49)* |
| **Bicarbonates (mmol/L)** | 25.6±5.5 (24.9-26.3) | 25.4±5.5 (24.7-26.1) | 26.3±5.5 (25.6-27)* | 26.9±17.5 (24.6-29.2) |
| **SaO_2_ (%)** | 94±4 (94-94) | 96±6 (95-97)* | 97±2 (96-97)* | 95±7 (94-96)* |
| **FiO_2_ (%)** | 64±20 (61-66) | 56±19 (53-58)* | 41±14 (39-43)* | 51±19 (49-53)* |
| **P_moy_ (cmH_2_O)** | 21±3 (21-22) | 21±3 (21-21) | 21±14 (19-23)* | 21±3 (20-21) |
| **P_plat_ (cmH_2_O)** | 29±4 (28-29) | 28±4 (28-29) | 27±4 (26-28)* | 28±4 (27-28)* |
| **PEEP_tot_ (cmH_2_O)** | 16±4 (15-16) | 16±3 (15-16) | 15±4 (15-16)* | 16±4 (15-16) |
| **RR (n/min)** | 25±6 (25-26) | 26±6 (25-26) | 26±6 (25-27)* | 26±6 (25-27) |
| **VT (mL/kg IBW)** | 7±1.6 (6.7-7.2) | 7±1.7 (6.8-7.2) | 7.9±11 (6.5-9.4)* | 7.1±1.7 (6.9-7.3) |
| **C_stat_ (mL/cmH_2_O)** | 39±16 (37-42) | 41±18 (38-44) | 44±16 (42-47)* | 43±17 (41-46)* |
| **C_dyn_ (mL/cmH_2_O)** | 39±21 (36-42) | 41±17 (38-43)* | 45±21 (42-49)* | 43±23 (39-46)* |
| **ΔP_dyn_ (cmH_2_O)** | 13.1±4.5 (12.4-13.7) | 12.2±3.8 (11.6-12.7)* | 11.5±3.9 (10.9-12.1)* | 12.2±4.5 (11.6-12.9)* |
| **R_aw_ (cmH_2_O/L/s)** | 14±5 (13-15) | 15±6 (14-16) | 14±6 (13-15) | 14±6 (13-15) |
| **Mechanical Power (J/min)** | 29.7±9.9 (28.3-31) | 30.7±10.3 (29.3-32.1) | 31.3±10.8 (29.8-32.8)* | 31.1±11 (29.6-32.6)* |
| **P_max_ (cmH_2_O)** | 36±6 (35-37) | 36±6 (35-37) | 35±6 (34-35)* | 36±6 (35-37) |
| **Vds (mL)** | 149±42 (143-155) | 153±43 (147-159) | 154±39 (148-159)* | 148±39 (142-153) |
| **S_III_ (%CO_2_/L)** | 7.9±5.7 (7.1-8.7) | 8.5±7.2 (7.5-9.4) | 8.5±14.3 (6.5-10.5) | 7±4.8 (6.3-7.6)* |
| **PetCO_2_ (mmHg)** | 39±9 (38-40) | 39±8 (38-40) | 36±8 (35-37)* | 35±7 (34-36)* |
| **FeCO_2_ (%)** | 5.4±1.2 (5.2-5.6) | 5.4±1.1 (5.2-5.6) | 5.1±1.1 (4.9-5.2)* | 5±1 (4.9-5.2)* |
| **VCO_2-min_ (mL/min)** | 236±68 (226-245) | 235±65 (226-244) | 235±63 (226-244) | 234±59 (226-242) |
| **PaO_2_/FiO_2_** | 125±50 (119-132) | 175±71 (166-184)* | 214±80 (204-225)* | 172±74 (162-181)* |
| **ΔP (cmH_2_O)** | 12.7±4.4 (12.1-13.4) | 12.4±4.6 (11.7-13.1) | 11.5±4 (10.9-12.1)* | 11.8±4.3 (11.1-12.4)* |
| **Oxygenation Index** | 19.1±7.3 (18.2-20.1) | 13.9±5.7 (13.2-14.6)* | 11.5±8.8 (10.3-12.6)* | 14.5±7.2 (13.6-15.5)* |
| **S_III-R_** | 2.67±1.63 (2.44-2.89) | 2.82±1.5 (2.62-3.02)* | 3.11±6.46 (2.22-4) | 2.41±1.22 (2.25-2.58)* |
| **Oxygen Stretch Index** | 12±6.5 (11-13) | 8.5±5.6 (7.6-9.4)* | 6.4±4.2 (5.7-7.1)* | 8.5±6.2 (7.5-9.5)* |
| **V_D_/V_T-phy_ (%)** | 34±8.3 (32.8-35.1) | 34.4±7.7 (33.3-35.4) | 33.9±7.9 (32.9-35) | 33±7.5 (32-34) |
| **P_(a-et)_CO_2_/PaCO_2_ (%)** | 23.7±16.8 (21.4-25.9) | 21.5±16.1 (19.3-23.6)* | 21.2±15.6 (19.1-23.3)* | 22.7±16.4 (20.5-24.8) |
| **V_E_/VCO_2-min_** | 67±144 (47.4-86.5) | 66.7±152 (46-87.3) | 68.1±148 (47.8-88.5)* | 56.5±71.5 (46.6-66.4)* |

Values are mean ± SD (95% Confidence Interval). * p < 0.05 compared to baseline value.

**Table S4. Factors associated with a marked increase in P_a_O_2_/F_i_O_2_** ratio. Univariate and multivariate analysis.

| **Parameter** | **ΔP_a_O_2_/F_i_O_2_**  **≤ 50%** | **ΔP_a_O_2_/F_i_O_2_**  **> 50%** | **p-univariate** | **Odds Ratio**  **(95% CI)** | **p-multivariate** |
| --- | --- | --- | --- | --- | --- |
| **ΔP_a_O_2_/F_i_O_2_** | 25.6 (-3;36.3) | 116 (88.1;172) | < 0.001 | - | - |
| **ΔP_plat_** | 0 (-6.9;3.4) | -2.8 (-12.1;3.2) | 0.358 | NIR | - |
| **ΔPEEP_tot_** | 0 (-15.1;0) | 0 (-9.9;12.7) | 0.109 | 0.99 (0.95;1.03) | 0.569 |
| **ΔRR_tot_** | 0 (-7;23.8) | 0 (-4.1;20) | 0.641 | NIR | - |
| **ΔVT** | 0 (-7.7;13.2) | 2.3 (-3.5;10.4) | 0.716 | NIR | - |
| **ΔC_stat_** | -1.2 (-10.9;10.6) | 16.7 (-6.5;33.4) | 0.052 | 1.04 (0.98;1.1) | 0.228 |
| **ΔΔP** | 3.3 (-6.3;12.9) | -11.1 (-21.7;4.2) | 0.022 | 1 (0.95;1.06) | 0.913 |
| **ΔM_pow_** | 10.9 (-3.7;48.4) | 10.8 (-15.3;31.6) | 0.44 | NIR | - |
| **ΔS_III_** | -9.4 (-3.04;14.3) | -16.5 (-36.9;30.7) | 1 | NIR | - |
| **ΔP_et_CO_2_** | -10.7 (-20.5;1.3) | -10.4 (-23.6;5.3) | 0.926 | NIR | - |
| **ΔVCO_2-min_** | 7.2 (-9.1;15.9) | -2.1 (-9.1;8.5) | 0.104 | 1 (0.99;1) | 0.761 |
| **ΔV_D_/V_T-phy_** | -8.2 (-22.8;4) | -1 (-13;9.5) | 0.092 | 1.05 (0.99;1.12) | 0.109 |
| **Session length** | 21.8 (18.7;24.2) | 21.5 (17.5;23.7) | 0.483 | NIR | - |

Values are differences (%) between datas of session 1 before PP and at end PP session (before return in SP) . Values are expressed with median (25^th^ ; 75^th^ quartile) and Odds Ratio (95% Confidence interval of OR). NIR: not included in regression. Parameters with p-value < 0.17 were included in multivariate analysis.. RR: respiratory rate; C_stat_: static compliance; ΔP: driving pressure; S_III_: phase 3 slope of volumetric capnography. Hosmer and Lemeshow Test chi^2^ (8df) = 7.893; p = 0.444.

**Table S5. Time (hours) to maximum effect between 0 to 24 hours (limit of record).**

| **Parameter** | **All sessions** | **Responder sessions** | **Session 1** |
| --- | --- | --- | --- |
| **V_D_/V_T-phy_** | 16 [13 ; 20] | 19 [16 ; 23] | 17 [13 ; 22] |
| **S_III_** | 18 [14 ; 21] | 18 [16 ; 22] | 18 [14 ; 21] |
| **P_et_CO_2_** | 16 [13 ; 19] | 16.5 [13 ; 19] | 17 [13 ; 21] |
| **C_dyn_** | 16 [14 ; 19] | 16 [14 ; 19] | 17 [14 ; 19] |
| **ΔP_dyn_** | 17 [14 ; 19] | 17 [14 ; 19] | 17 [15 ; 21] |

**Values are median mean of maximum effect per patient with interquartile range 25^th^ – 75^th^ .**

**Table S6. Univariate analysis of patients’ characteristics at inclusion during ICU and hospital stays between dead and survivors.**

|  | **Dead** | **Survivors** | **p-value** |
| --- | --- | --- | --- |
| **Inclusion** | | | |
| **N (%)** | 39 (37.9) | 64 (62.1) | - |
| **Age (years)** | 67.6±12.6 (63.6-71.5) | 56.7±12.1 (53.7-59.6) | < 0.001 |
| **Male gender (n)** | 25 (64.1) | 48 (75) | 0.269 |
| **BMI (kg/m^2^)** | 28.3±6.3 (26.3-30.3) | 29.8±7.7 (27.9-31.7) | 0.428 |
| **SAPS2** | 63±17 (57-68) | 49±15 (45-52) | < 0.001 |
| **SOFA** | 10±4 (9-12) | 9±3 (8-10) | 0.062 |
| **ICU stay** | | | |
| **Prone position sessions (n per patient)** | 2.3±2.2 (1.6-3) | 2.2±1.5 (1.8-2.6) | 0.540 |
| **Length of sessions (hours)** | 21.1±6.5 (19.1-23.2) | 20.8±3 (20.1-21.6) | 0.719 |
| **Delay between sessions (hours)** | 67.4±124 (1.41-134) | 62.8±88.1 (33-92.6) | 0.835 |
| **Invasive mechanical ventilation (days)** | 15±11 (11.5-18.4) | 18.7±10.7 (16.1-21.3) | 0.038 |
| **Non-invasive ventilation (n)** | 23 (59) | 50 (78.1) | 0.046 |
| **Non-invasive ventilation (days)** | 2.3±1.7 (1.6-2.9) | 3.6±2.9 (2.8-4.4) | 0.036 |
| **Vasopressor (n)** | 39 (100) | 62 (96.9) | 0.525 |
| **Vasopressor duration (days)** | 10.9±8.8 (8.2-13.7) | 7.9±5.9 (6.4-9.3) | 0.063 |
| **Tracheostomy (n)** | 0 | 10 (15.6) | 0.012 |
| **RRT (n)** | 16 (41) | 14 (21.9) | 0.046 |
| **Corticosteroids (n)** | 34 (87.1) | 34 (53.1) | < 0.001 |
| **NMBA (n)** | 37 (92.5) | 60 (95.2) | 0.926 |
| **Complications and outcomes** | | | |
| **Pneumothorax (n)** | 1 (2.6) | 1 (1.6) | 1 |
| **Ventilation-associated pneumonia (n)** | 16 (41) | 28 (43.8) | 0.839 |
| **ICU Lenght of stay (days)** | 16.5±11.2 (13-20) | 25.8±14.2 (22.3-29.3) | < 0.001 |
| **Hospital Lenght of stay (days)** | 18.8±12.8 (14.8-22.8) | 32.2±14.8 (28.6-35.9) | < 0.001 |
| **ICU Deaths with ARDS (n)** | 34 (87.1) | 0 | - |
| **ICU Deaths (n)** | 38 (97.4) | 0 | - |
| **Respiratory characteristics before the first PP session** | | | |
| **pH** | 7.24±0.1 (7.2-7.29) | 7.27±0.1 (7.24-7.29) | 0.517 |
| **PaO_2_ (mmHg)** | 77±39 (65-89) | 77±28 (70-84) | 0.228 |
| **PaCO_2_ (mmHg)** | 53±12 (49-56) | 55±13 (52-58) | 0.491 |
| **Bicarbonates (mmol/L)** | 22.4±5.3 (20.7-24.1) | 24.9±5.4 (23.5-26.2) | 0.036 |
| **SaO_2_ (%)** | 94±3 (93-95) | 94±3 (93-95) | 0.43 |
| **FiO_2_ (%)** | 66±22 (59-73) | 65±21 (59-70) | 0.854 |
| **P_moy_ (cmH_2_O)** | 21±4 (20-22) | 21±3 (21-22) | 0.433 |
| **P_plat_ (cmH_2_O)** | 29±4 (27-30) | 29±4 (27-30) | 0.702 |
| **PEEP_tot_ (cmH_2_O)** | 15±4 (14-16) | 16±3 (16-17) | 0.138 |
| **RR (n/min)** | 26±7 (24-29) | 23±6 (22-25) | 0.025 |
| **VT (mL/kg IBW)** | 6.7±1.2 (6.3-7.1) | 7.1±1.7 (6.7-7.5) | 0.323 |
| **C_stat_ (mL/cmH_2_O)** | 33±14 (28-38) | 43±16 (39-48) | 0.009 |
| **C_dyn_ (mL/cmH_2_O)** | 31±16 (25-37) | 41±17 (36-45) | 0.005 |
| **ΔP_dyn_ (cmH_2_O)** | 15.4±5.1 (13.6-17.2) | 12.8±4.3 (11.6-13.9) | 0.014 |
| **R_aw_ (cmH_2_O/L/s)** | 14±6 (12-16) | 14±5 (12-15) | 0.791 |
| **Mechanical Power (J/min)** | 29.9±10.4 (26.2-33.6) | 29±9.1 (26.5-31.5) | 0.806 |
| **P_max_ (cmH_2_O)** | 38±7 (35-40) | 36±6 (34-37) | 0.153 |
| **Vds (mL)** | 142±32 (131-153) | 169±51 (156-183) | 0.009 |
| **S_III_ (%CO_2_/L)** | 10.6±8.3 (7.8-13.4) | 8.52±5.78 (7.04-10.01) | 0.572 |
| **PetCO_2_ (mmHg)** | 37±9 (35-40) | 43±9 (41-46) | 0.002 |
| **FeCO_2_ (%)** | 5.23±1.24 (4.8-5.65) | 6.05±1.27 (5.72-6.38) | 0.004 |
| **VCO_2-min_ (mL/min)** | 185±81 (157-212) | 250±53 (236-263) | < 0.001 |
| **PaO_2_/FiO_2_** | 128±63 (109-148) | 129±46 (117-140) | 0.659 |
| **ΔP (cmH_2_O)** | 13.7±4.5 (12.1-15.4) | 12±3.5 (11-13) | 0.096 |
| **Oxygenation Index** | 18.9±7.8 (16.4-21.4) | 18.7±6.7 (17.1-20.4) | 0.953 |
| **S_III-R_** | 3±2.03 (2.31-3.69) | 3.07±1.87 (2.59-3.55) | 0.583 |
| **Oxygen Stretch Index** | 13.4±7.5 (10.5-16.2) | 11.1±5.5 (9.5-12.6) | 0.249 |
| **V_D_/V_T-phy_ (%)** | 35.5±9 (32.5-38.6) | 37.2±8.3 (35.1-39.3) | 0.37 |
| **P_(a-et)_CO_2_/PaCO_2_ (%)** | 26.9±16.1 (21.6-32.2) | 19.1±16.2 (15-23.2) | 0.033 |
| **V_E_/VCO_2-min_** | 110±214 (36.5-183) | 42±8.7 (39.8-44.2) | < 0.001 |

Values are n (%) and mean ± SD (95% Confidence Interval).

**Table S7. Multivariate analysis of Δchange, before PP-before SP, in respiratory parameters between dead and survivors during the first PP session.**

| **Parameters** | **Dead** | **Survivors** | **P-univariate** | **Odds Ratio**  **(95% CI)** | **P-multivariate** |
| --- | --- | --- | --- | --- | --- |
| **pH** | 0.81 (0.14 ; 2.33) | 1.09 (0.55 ; 2.04) | 0.608 | NIR |  |
| **PaO_2_ (mmHg)** | 8.82 (-5.95 ; 41.5) | 12.9 (-2.63 ; 25) | 0.674 | NIR |  |
| **PaCO_2_ (mmHg)** | -13.3 (-25.9 ; -0.53) | -12.5 (-29.4 ; -3.85) | 0.506 | NIR |  |
| **Bicarbonates (mmol/L)** | 3.92 (-7.72 ; 12.6) | 1.56 (-8.7 ; 15.5) | 0.917 | NIR |  |
| **SaO_2_ (%)** | 2.72 (-0.68 ; 4.98) | 2.39 (0.86 ; 4.76) | 0.84 | NIR |  |
| **FiO_2_ (%)** | -40 (-50 ; -18.3) | -40 (-50 ; -25) | 0.438 | NIR |  |
| **P_moy_ (cmH_2_O)** | -5 (-9.52 ; 8.35) | 0 (-13.4 ; 5) | 0.651 | NIR |  |
| **P_plat_ (cmH_2_O)** | -4.35 (-12.1 ; 3.31) | -1.78 (-14.3 ; 1.69) | 0.715 | NIR |  |
| **PEEP_tot_ (cmH_2_O)** | 0 (-14.3 ; 12.7) | 0 (-12.5 ; 6.07) | 0.276 | NIR |  |
| **RR (n/min)** | 0 (-9.95 ; 10.5) | 6.67 (0 ; 20.5) | 0.09 | 0.99 (0.97 ; 1) | 0.151 |
| **VT (mL/kg IBW)** | 3 (-2.98 ; 13.8) | 1.09 (-7.05 ; 10) | 0.662 | NIR |  |
| **C_stat_ (mL/cmH_2_O)** | 15.9 (3.5 ; 32.1) | 7.5 (-10.3 ; 31.9) | 0.436 | NIR |  |
| **C_dyn_ (mL/cmH_2_O)** | 28.8 (3.52 ; 55.1) | 17.9 (-0.89 ; 35.2) | 0.097 | NIR |  |
| **ΔP_dyn_ (cmH_2_O)** | -19.6 (-30.3 ; -3.15) | -9.47 (-23.4 ; -0.91) | 0.121 | 0.99 (0.98 ; 1) | 0.287 |
| **R_aw_ (cmH_2_O/L/s)** | 13.2 (-11.1 ; 45.8) | 0 (-21.1 ; 10) | 0.123 | 1 (0.99 ; 1) | 0.323 |
| **Mechanical Power (J/min)** | 0.22 (-14 ; 14.3) | 23.2 (-12.2 ; 48.1) | 0.097 | NIR |  |
| **P_max_ (cmH_2_O)** | 0 (-14.5 ; 5.3) | -2.27 (-12.3 ; 6.17) | 0.968 | NIR |  |
| **Vds (mL)** | 4.27 (-0.88 ; 14.8) | -3.95 (-14.8 ; 5.26) | 0.006 | NIR |  |
| **S_III_ (%CO_2_/L)** | -10.2 (-30.7 ; 50.6) | -17.4 (-31 ; 7.76) | 0.293 | NIR |  |
| **PetCO_2_ (mmHg)** | -6.49 (-23 ; 7.47) | -10.7 (-22.4 ; -0.52) | 0.312 | NIR |  |
| **FeCO_2_ (%)** | -1.96 (-14.3 ; 12.5) | -11.1 (-21.2 ; 0) | 0.146 | NIR |  |
| **VCO_2-min_ (mL/min)** | 8.6 (-0.6 ; 18.5) | -4.64 (-11 ; 4.48) | 0.005 | 1.04 (1.04 ; 1.07) | 0.015 |
| **PaO_2_/FiO_2_** | 97.8 (32 ; 153) | 88.1 (50 ; 126) | 0.933 | NIR |  |
| **ΔP (cmH_2_O)** | -11.1 (-19.4 ; 8.36) | -9.09 (-15.4 ; 6.67) | 0.71 | NIR |  |
| **Oxygenation Index** | -49.9 (-60.1 ; -18.3) | -49.5 (-58.7 ; -42.5) | 0.469 | NIR |  |
| **S_III-R_** | 9.47 (-35.4 ; 47) | -8 (-25.3 ; 20.4) | 0.309 | NIR |  |
| **Oxygen Stretch Index** | -55.4 (-72.1 ; -32) | -51.3 (-67 ; -37.3) | 0.651 | NIR |  |
| **V_D_/V_T-phy_ (%)** | 1.63 (-12.6 ; 17.6) | -3.66 (-17 ; 5.08) | 0.133 | 1.03 (1 ; 1.05) | 0.031 |
| **P_(a-et)_CO_2_/PaCO_2_ (%)** | -7.69 (-26.1 ; 15.9) | -19.2 (-64.5 ; 22.8) | 0.149 | NIR |  |
| **V_E_/VCO_2-min_** | -7.15 (-17.6 ; 11.8) | 14.1 (-1.76 ; 31) | 0.003 | NIR |  |

Values are Δchange in % (95% Confidence interval of Δchange). NIR : Not included In Regression. Hosmer and Lemeshow Test Chi2 (8 df) = 10.43 ; p = 0.236

**Table S8. Multivariate analysis of Δchange, before PP-2 hours after SP, in respiratory parameters between dea**d and survivors during the first PP session.

| **Parameters** | **Dead** | **Survivors** | **P-value** | **Odds Ratio**  **(95% CI)** | **P-multivariate** |
| --- | --- | --- | --- | --- | --- |
| **pH** | 1.08 (-0.14 ; 1.85) | 1.3 (0.27 ; 2.17) | 0.593 | NIR |  |
| **PaO_2_ (mmHg)** | 4.23 (-10.4 ; 29.5) | 0 (-17.5 ; 27) | 0.441 | NIR |  |
| **PaCO_2_ (mmHg)** | -13.6 (-24.6 ; 0.56) | -20.6 (-28.4 ; -5.9) | 0.13 | NIR |  |
| **Bicarbonates (mmol/L)** | 1.81 (-7.96 ; 11) | 0 (-10.8 ; 10.6) | 0.575 | NIR |  |
| **SaO_2_ (%)** | 2.56 (-0.26 ; 4.24) | 1.5 (-1.04 ; 3.87) | 0.402 | NIR |  |
| **FiO_2_ (%)** | -20 (-41.3 ; -6.61) | -30 (-50 ; -16.7) | 0.35 | NIR |  |
| **P_moy_ (cmH_2_O)** | -3.94 (-10.3 ; 8.19) | -5 (-15 ; 4.88) | 0.211 | NIR |  |
| **P_plat_ (cmH_2_O)** | 2.33 (-6.51 ; 9.94) | -3.68 (-12.5 ; 0) | 0.082 | 1.03 (0.98 ; 1.09) | 0.254 |
| **PEEP_tot_ (cmH_2_O)** | 0 (-11.5 ; 18.6) | 0 (-12.5 ; 6.07) | 0.222 | NIR |  |
| **RR (n/min)** | 0 (-7.59 ; 12.4) | 7.14 (0 ; 20) | 0.127 | 0.99 (0.95 ; 1.02) | 0.441 |
| **VT (mL/kg IBW)** | 3.95 (-6.34 ; 12.6) | 0.91 (-6.52 ; 8.56) | 0.616 | NIR |  |
| **C_stat_ (mL/cmH_2_O)** | 3.53 (-4.82 ; 14.9) | 9.66 (-1.25 ; 31.2) | 0.229 | NIR |  |
| **C_dyn_ (mL/cmH_2_O)** | 16 (0 ; 31.3) | 7.69 (-1.88 ; 23.6) | 0.398 | NIR |  |
| **ΔP_dyn_ (cmH_2_O)** | -12.9 (-21.6 ; 1.23) | -8.73 (-21.8 ; 4.07) | 0.376 | NIR |  |
| **R_aw_ (cmH_2_O/L/s)** | 0 (-16.7 ; 8.33) | 0 (-25.9 ; 16.7) | 0.944 | NIR |  |
| **Mechanical Power (J/min)** | -1.53 (-23.5 ; 24) | 14.8 (-9.55 ; 33) | 0.189 | NIR |  |
| **P_max_ (cmH_2_O)** | -5.41 (-10.5 ; 5.71) | -2.63 (-8.83 ; 7.2) | 0.513 | NIR |  |
| **Vds (mL)** | 0.81 (-3.96 ; 8.63) | -9.71 (-19.9 ; 2.36) | 0.009 | NIR |  |
| **S_III_ (%CO_2_/L)** | -17.4 (-41.7 ; -0.2) | -21 (-39.5 ; 0.85) | 0.76 | NIR |  |
| **PetCO_2_ (mmHg)** | -14.7 (-23.1 ; 0) | -17.1 (-23 ; -4.76) | 0.648 | NIR |  |
| **FeCO_2_ (%)** | -11.5 (-20.7 ; 3.1) | -16.4 (-22.3 ; -4.12) | 0.239 | NIR |  |
| **VCO_2-min_ (mL/min)** | 4.19 (-9.67 ; 14.9) | -7.42 (-14 ; 7.14) | 0.06 | 1 (0.99 ; 1.01) | 0.594 |
| **PaO_2_/FiO_2_** | 45.8 (10.6 ; 79.7) | 41.7 (17.9 ; 88) | 0.713 | NIR |  |
| **ΔP (cmH_2_O)** | -8.2 (-13.8 ; 6.23) | -8.39 (-20.8 ; 5.76) | 0.595 | NIR |  |
| **Oxygenation Index** | -32.8 (-46.4 ; -10.5) | -38.8 (-50.7 ; -14.7) | 0.34 | NIR |  |
| **S_III-R_** | -6.09 (-44.7 ; 8.31) | -18.3 (-35.9 ; -0.06) | 0.37 | NIR |  |
| **Oxygen Stretch Index** | -39.6 (-57.1 ; -15.8) | -35.5 (-51.8 ; -20.3) | 0.742 | NIR |  |
| **V_D_/V_T-phy_ (%)** | -0.59 (-11.2 ; 9.87) | -9 (-20 ; 0.27) | 0.036 | 1.02 (0.99 ; 1.04) | 0.193 |
| **P_(a-et)_CO_2_/PaCO_2_ (%)** | -5.65 (-43.8 ; 37.6) | -6.78 (-58.6 ; 32.4) | 0.428 | NIR |  |
| **V_E_/VCO_2-min_** | -3.32 (-22.2 ; 16.1) | 14.7 (-2.56 ; 30.4) | 0.011 | NIR |  |

Values are Δchange in % (95% Confidence interval of Δchange). NIR : Not included In Regression. Hosmer and Lemeshow Test Chi2 (8 df) = 9.565 ; p = 0.297

**Table S9.** Lung mechanics and outcome regarding first and average PP sessions.

|  | **Δchange before PP-before SP** | |  | **Δchange before PP-after SP** | | | |  |
| --- | --- | --- | --- | --- | --- | --- | --- | --- |
| **Parameters** | Dead | Survivors | P-value | Dead | | Survivors | P-value | |
| **PP Session 1** | | | | | | | | |
| **pH** | 0.81 (0.14 ; 2.33) | 1.09 (0.55 ; 2.04) | 0.608 | 1.08 (-0.14 ; 1.85) | | 1.3 (0.27 ; 2.17) | 0.593 | |
| **P_a_CO_2_ (mmHg)** | -13.3 (-25.9 ; -0.53) | -12.5 (-29.4 ; -3.85) | 0.506 | -13.6 (-24.6 ; 0.56) | | -20.6 (-28.4 ; -5.9) | 0.13 | |
| **F_i_O_2_ (%)** | -40 (-50 ; -18.3) | -40 (-50 ; -25) | 0.438 | -20 (-41.3 ; -6.61) | | -30 (-50 ; -16.7) | 0.35 | |
| **P_plat_ (cmH_2_O)** | -4.35 (-12.1 ; 3.31) | -1.78 (-14.3 ; 1.69) | 0.715 | 2.33 (-6.51 ; 9.94) | | -3.68 (-12.5 ; 0) | 0.082 | |
| **PEEP_tot_ (cmH_2_O)** | 0 (-14.3 ; 12.7) | 0 (-12.5 ; 6.07) | 0.276 | 0 (-11.5 ; 18.6) | | 0 (-12.5 ; 6.07) | 0.222 | |
| **RR (cycles/min)** | 0 (-9.95 ; 10.5) | 6.67 (0 ; 20.5) | 0.09 | 0 (-7.59 ; 12.4) | | 7.14 (0 ; 20) | 0.127 | |
| **VT_e_ (mL/kg PBW)** | 3 (-2.98 ; 13.8) | 1.09 (-7.05 ; 10) | 0.662 | 3.95 (-6.34 ; 12.6) | | 0.91 (-6.52 ; 8.56) | 0.616 | |
| **C_stat_ (mL/cmH_2_O)** | 15.9 (3.5 ; 32.1) | 7.5 (-10.3 ; 31.9) | 0.436 | 3.53 (-4.82 ; 14.9) | | 9.66 (-1.25 ; 31.2) | 0.229 | |
| **S_III_ (%CO_2_/L)** | -10.2 (-30.7 ; 50.6) | -17.4 (-31 ; 7.76) | 0.293 | -17.4 (-41.7 ; -0.2) | | -21 (-39.5 ; 0.85) | 0.76 | |
| **P_et_CO_2_ (mmHg)** | -6.49 (-23 ; 7.47) | -10.7 (-22.4 ; -0.52) | 0.312 | -14.7 (-23.1 ; 0) | | -17.1 (-23 ; -4.76) | 0.648 | |
| **VCO_2-min_ (mL/min)** | 8.6 (-0.6 ; 18.5) | -4.64 (-11 ; 4.48) | 0.005 | 4.19 (-9.67 ; 14.9) | | -7.42 (-14 ; 7.14) | 0.06 | |
| **P_a_O_2_/F_i_O_2_** | 97.8 (32 ; 153) | 88.1 (50 ; 126) | 0.933 | 45.8 (10.6 ; 79.7) | | 41.7 (17.9 ; 88) | 0.713 | |
| **ΔP (cmH_2_O)** | -11.1 (-19.4 ; 8.36) | -9.09 (-15.4 ; 6.67) | 0.71 | -8.2 (-13.8 ; 6.23) | | -8.39 (-20.8 ; 5.76) | 0.595 | |
| **V_D_/V_Tphy_ (%)** | 1.63 (-12.6 ; 17.6) | -3.66 (-17 ; 5.08) | 0.133 | -0.59 (-11.2 ; 9.87) | | -9 (-20 ; 0.27) | 0.036 | |
| **P_(a-et)_CO_2_/P_a_CO_2_ (%)** | -7.69 (-26.1 ; 15.9) | -19.2 (-64.5 ; 22.8) | 0.149 | -5.65 (-43.8 ; 37.6) | | -6.78 (-58.6 ; 32.4) | 0.428 | |
| **V_E_/VCO_2-min_** | -7.15 (-17.6 ; 11.8) | 14.1 (-1.76 ; 31) | 0.003 | -3.32 (-22.2 ; 16.1) | | 14.7 (-2.56 ; 30.4) | 0.011 | |
| **Average PP sessions per patient** | | | | | | | | |
| **pH** | 0.62 (0.1 ; 1.55) | 0.96 (0.47 ; 1.44) | 0.389 | 0.56 (-0.19 ; 1.08) | 0.89 (0.41 ; 1.55) | | 0.049 | |
| **P_a_CO_2_ (mmHg)** | -12.8 (-23.2 ; 0.66) | -8.58 (-19.6 ; 1.52) | 0.741 | -11.9 (-17.7 ; 0.56) | -11.5 (-22.5 ; -2.94) | | 0.359 | |
| **F_i_O_2_ (%)** | -34.3 (-46.3 ; -24.5) | -37.5 (-44.9 ; -25) | 0.665 | -16.7 (-39.6 ; -3.87) | -25 (-37 ; -11.8) | | 0.239 | |
| **P_plat_ (cmH_2_O)** | -0.39 (-12.1 ; 3.92) | -4.51 (-8.25 ; 0) | 0.601 | -1.54 (-7.6 ; 4.79) | -2.37 (-7.99 ; 8.22) | | 0.586 | |
| **PEEP_tot_ (cmH_2_O)** | 0 (-14.3 ; 7.27) | 0 (-9.9 ; 3.6) | 0.687 | -1.72 (-11.1 ; 12.1) | 0 (-7.89 ; 8.22) | | 0.668 | |
| **RR (cycles/min)** | 0 (-3.95 ; 9.32) | 2.14 (-6.75 ; 13.2) | 0.795 | 0 (-6.56 ; 6.02) | 3.43 (-4.4 ; 10.3) | | 0.171 | |
| **VT_e_ (mL/kg PBW)** | 4.72 (-2.15 ; 12.6) | 1.2 (-5.41 ; 7.25) | 0.259 | 4.46 (-1.9 ; 11.7) | 1.46 (-4.97 ; 6.75) | | 0.235 | |
| **C_stat_ (mL/cmH_2_O)** | 19.3 (11.9 ; 27.7) | 10.8 (-2.46 ; 28.5) | 0.256 | 7.88 (0.54 ; 14.2) | 7.76 (-0.73 ; 32.5) | | 0.605 | |
| **S_III_ (%CO_2_/L)** | 0 (-15.4 ; 41.2) | -6.35 (-30.3 ; 11.5) | 0.172 | -3.35 (-19.3 ; 5.7) | -19.9 (-32 ; 5.26) | | 0.163 | |
| **P_et_CO_2_ (mmHg)** | -7.95 (-16.4 ; 4.14) | -7.32 (-15.7 ; 2.56) | 0.965 | -9.09 (-19.3 ; -1.94) | -8.54 (-18.9 ; -3.94) | | 0.936 | |
| **VCO_2-min_ (mL/min)** | 0 (-7.43 ; 11.1) | -3.15 (-9.31 ; 5.81) | 0.257 | 3.26 (-6.78 ; 11) | -3.25 (-11.8 ; 2.9) | | 0.058 | |
| **P_a_O_2_/F_i_O_2_** | 80.8 (17.3 ; 120) | 78.5 (36.9 ; 109) | 0.752 | 27.8 (4.69 ; 56.6) | 38.3 (15 ; 88.5) | | 0.225 | |
| **ΔP (cmH_2_O)** | -12.2 (-17.1 ; 0.48) | -8.33 (-21.2 ; 6.67) | 0.989 | -5.66 (-13.9 ; 0) | -4.83 (-22.7 ; 5.42) | | 0.768 | |
| **V_D_/V_T-phy_ (%)** | 2.58 (-7.32 ; 10.1) | 0.23 (-11.3 ; 8.32) | 0.214 | -1 (-7.62 ; 9.03) | -3.98 (-11.5 ; 6.16) | | 0.136 | |
| **P_(a-et)_CO_2_/P_a_CO_2_ (%)** | -13.4 (-24.3 ; 10.7) | -8.82 (-45.5 ; 12) | 0.58 | -9.19 (-23.4 ; 4.1) | 1.41 (-44.3 ; 24.2) | | 0.438 | |
| **V_E_/VCO_2-min_** | 2.67 (-8.08 ; 15) | 5.56 (-6.43 ; 24.1) | 0.308 | 2.19 (-9.83 ; 17.49) | 5.97 (-0.08 ; 28.1) | | 0.101 | |

Univariate analysis, between the dead patients and the survivors, according to the evolution of the respiratory parameters before prone position, before returning to a supine position and after returning to a supine position, during the first prone position session and during the average of the prone position sessions per patient. Values are expressed with median (25^th^ ; 75^th^ quartile). RR: respiratory rate; C_stat_: static compliance; ΔP: driving pressure; S_III_: phase 3 slope of volumetric capnography; V_E_: minute ventilation.

**Table S10. Multivariate analysis of Δchange, before PP-before** SP, in respiratory parameters between dead and survivors during the average PP session per patient.

| **Parameters** | **Dead** | **Survivors** | **P-value** | **Odds Ratio**  **(95% CI)** | **P-multivariate** |
| --- | --- | --- | --- | --- | --- |
| **pH** | 0.62 (0.1 ; 1.55) | 0.96 (0.47 ; 1.44) | 0.389 | NIR |  |
| **PaO_2_ (mmHg)** | 13.3 (-2.51 ; 22.9) | 15.9 (0.11 ; 26.9) | 0.719 | NIR |  |
| **PaCO_2_ (mmHg)** | -12.8 (-23.2 ; 0.66) | -8.58 (-19.6 ; 1.52) | 0.741 | NIR |  |
| **Bicarbonates (mmol/L)** | 2.09 (-4.91 ; 5.15) | 5.51 (-1.71 ; 11.8) | 0.092 | 0.96 (0.94 ; 1.01) | 0.1 |
| **SaO_2_ (%)** | 2.68 (0.19 ; 4.84) | 2.28 (0.91 ; 4.85) | 0.779 | NIR |  |
| **FiO_2_ (%)** | -34.3 (-46.3 ; -24.5) | -37.5 (-44.9 ; -25) | 0.665 | NIR |  |
| **P_moy_ (cmH_2_O)** | 0 (-8.48 ; 7.24) | -1.09 (-9.68 ; 4.82) | 0.66 | NIR |  |
| **P_plat_ (cmH_2_O)** | -0.39 (-12.1 ; 3.92) | -4.51 (-8.25 ; 0) | 0.601 | NIR |  |
| **PEEP_tot_ (cmH_2_O)** | 0 (-14.3 ; 7.27) | 0 (-9.9 ; 3.6) | 0.687 | NIR |  |
| **RR (n/min)** | 0 (-3.95 ; 9.32) | 2.14 (-6.75 ; 13.2) | 0.795 | NIR |  |
| **VT (mL/kg IBW)** | 4.72 (-2.15 ; 12.6) | 1.2 (-5.41 ; 7.25) | 0.259 | NIR |  |
| **C_stat_ (mL/cmH_2_O)** | 19.3 (11.9 ; 27.7) | 10.8 (-2.46 ; 28.5) | 0.256 | NIR |  |
| **C_dyn_ (mL/cmH_2_O)** | 18.9 (2.73 ; 41) | 16.6 (2.19 ; 26.9) | 0.447 | NIR |  |
| **ΔP_dyn_ (cmH_2_O)** | -10.4 (-26.8 ; -2.91) | -10.5 (-19.9 ; -2.21) | 0.682 | NIR |  |
| **R_aw_ (cmH_2_O/L/s)** | 9.72 (-11.1 ; 33.3) | 0 (-18.6 ; 11.8) | 0.094 | 1.02 (1 ; 1.04) | 0.046 |
| **Mechanical Power (J/min)** | 4.5 (-6.51 ; 21.9) | 9.26 (-12.3 ; 27.4) | 0.612 | NIR |  |
| **P_max_ (cmH_2_O)** | 0 (-9.91 ; 4.44) | -4.09 (-10.8 ; 3.24) | 0.504 | NIR |  |
| **Vds (mL)** | 4.27 (0 ; 17) | 0.72 (-7.46 ; 8.04) | 0.021 | 1.03 (0.99 ; 1.07) | 0.176 |
| **S_III_ (%CO_2_/L)** | 0 (-15.4 ; 41.2) | -6.35 (-30.3 ; 11.5) | 0.172 | NIR |  |
| **PetCO_2_ (mmHg)** | -7.95 (-16.4 ; 4.14) | -7.32 (-15.7 ; 2.56) | 0.965 | NIR |  |
| **FeCO_2_ (%)** | -5.44 (-13.3 ; 7.69) | -6.67 (-15.2 ; 4.18) | 0.546 | NIR |  |
| **VCO_2-min_ (mL/min)** | 0 (-7.43 ; 11.1) | -3.15 (-9.31 ; 5.81) | 0.257 | NIR |  |
| **PaO_2_/FiO_2_** | 80.8 (17.3 ; 120) | 78.5 (36.9 ; 109) | 0.752 | NIR |  |
| **ΔP (cmH_2_O)** | -12.2 (-17.1 ; 0.48) | -8.33 (-21.2 ; 6.67) | 0.989 | NIR |  |
| **Oxygenation Index** | -42.5 (-56.7 ; -16.7) | -45.1 (-56.4 ; -32.9) | 0.496 | NIR |  |
| **S_III-R_** | 9.47 (-9.52 ; 43.5) | -1.82 (-21.2 ; 24.4) | 0.172 | NIR |  |
| **Oxygen Stretch Index** | -49.4 (-68.4 ; -37.8) | -46.6 (-63.3 ; -32.7) | 0.456 | NIR |  |
| **V_D_/V_T-phy_ (%)** | 2.58 (-7.32 ; 10.1) | 0.23 (-11.3 ; 8.32) | 0.214 | NIR |  |
| **P_(a-et)_CO_2_/PaCO_2_ (%)** | -13.4 (-24.3 ; 10.7) | -8.82 (-45.5 ; 12) | 0.58 | NIR |  |
| **V_E_/VCO_2-min_** | 2.67 (-8.08 ; 15) | 5.56 (-6.43 ; 24.1) | 0.308 | NIR |  |

Values are Δchange in % (95% Confidence interval of Δchange). NIR : Not included In Regression. Hosmer and Lemeshow Test Chi2 (8 df) = 2.683 ; p = 0.953

**Table S11. Multivariate analysis of Δchange, before PP-2 hours after SP, in respiratory parameters between dead and survivors d**uring the average PP session per patient.

| **Parameters** | **Dead** | **Survivors** | **P-value** | **Odds Ratio**  **(95% CI)** | **P-multivariate** |
| --- | --- | --- | --- | --- | --- |
| **pH** | 0.56 (-0.19 ; 1.08) | 0.89 (0.41 ; 1.55) | 0.049 | 0.84 (0.48 ; 1.45) | 0.524 |
| **PaO_2_ (mmHg)** | 3.36 (-8.96 ; 16.2) | 1.52 (-9.33 ; 26.8) | 0.977 | NIR |  |
| **PaCO_2_ (mmHg)** | -11.9 (-17.7 ; 0.56) | -11.5 (-22.5 ; -2.94) | 0.359 | NIR |  |
| **Bicarbonates (mmol/L)** | -0.65 (-7.95 ; 3.71) | 1.47 (-5.38 ; 10.4) | 0.111 | 0.98 (0.95 ; 1.02) | 0.351 |
| **SaO_2_ (%)** | 1.36 (-0.48 ; 3.12) | 1.49 (-0.32 ; 3.61) | 0.817 | NIR |  |
| **FiO_2_ (%)** | -16.7 (-39.6 ; -3.87) | -25 (-37 ; -11.8) | 0.239 | NIR |  |
| **P_moy_ (cmH_2_O)** | -2.44 (-9.55 ; 5.24) | -1.69 (-8.74 ; 5.28) | 0.74 | NIR |  |
| **P_plat_ (cmH_2_O)** | -1.54 (-7.6 ; 4.79) | -2.37 (-7.99 ; 8.22) | 0.586 | NIR |  |
| **PEEP_tot_ (cmH_2_O)** | -1.72 (-11.1 ; 12.1) | 0 (-7.89 ; 8.22) | 0.668 | NIR |  |
| **RR (n/min)** | 0 (-6.56 ; 6.02) | 3.43 (-4.4 ; 10.3) | 0.171 | NIR |  |
| **VT (mL/kg IBW)** | 4.46 (-1.9 ; 11.7) | 1.46 (-4.97 ; 6.75) | 0.235 | NIR |  |
| **C_stat_ (mL/cmH_2_O)** | 7.88 (0.54 ; 14.2) | 7.76 (-0.73 ; 32.5) | 0.605 | NIR |  |
| **C_dyn_ (mL/cmH_2_O)** | 13.3 (-2.82 ; 30.2) | 7.35 (-2.18 ; 23.8) | 0.366 | NIR |  |
| **ΔP_dyn_ (cmH_2_O)** | -11 (-19.6 ; 7.98) | -7.27 (-20.7 ; 4.8) | 0.74 | NIR |  |
| **R_aw_ (cmH_2_O/L/s)** | 0 (-11.3 ; 11.3) | 1.06 (-15.7 ; 11.4) | 0.881 | NIR |  |
| **Mechanical Power (J/min)** | 1.56 (-14.3 ; 13.2) | 9.5 (-5.38 ; 22.2) | 0.171 | NIR |  |
| **P_max_ (cmH_2_O)** | -3.93 (-9.7 ; 5.88) | -0.29 (-7.5 ; 7.61) | 0.505 | NIR |  |
| **Vds (mL)** | 2.22 (-3.43 ; 16.8) | -2 (-8.23 ; 4.98) | 0.03 | NIR |  |
| **S_III_ (%CO_2_/L)** | -3.35 (-19.3 ; 5.7) | -19.9 (-32 ; 5.26) | 0.163 | NIR |  |
| **PetCO_2_ (mmHg)** | -9.09 (-19.3 ; -1.94) | -8.54 (-18.9 ; -3.94) | 0.936 | NIR |  |
| **FeCO_2_ (%)** | -5.38 (-13.9 ; 2.38) | -7.77 (-16.7 ; -2.3) | 0.222 | NIR |  |
| **VCO_2-min_ (mL/min)** | 3.26 (-6.78 ; 11) | -3.25 (-11.8 ; 2.9) | 0.058 | 1.02 (1 ; 1.05) | 0.092 |
| **PaO_2_/FiO_2_** | 27.8 (4.69 ; 56.6) | 38.3 (15 ; 88.5) | 0.225 | NIR |  |
| **ΔP (cmH_2_O)** | -5.66 (-13.9 ; 0) | -4.83 (-22.7 ; 5.42) | 0.768 | NIR |  |
| **Oxygenation Index** | -25.4 (-40.1 ; 0.57) | -26.4 (-47.4 ; -9.04) | 0.261 | NIR |  |
| **S_III-R_** | -0.16 (-12.8 ; 15) | -14 (-25.9 ; 3.09) | 0.028 | 1 (0.99 ; 1.02) | 0.462 |
| **Oxygen Stretch Index** | -30.5 (-55.3 ; -1.45) | -31.7 (-47.3 ; 11.3) | 0.984 | NIR |  |
| **V_D_/V_T-phy_ (%)** | -1 (-7.62 ; 9.03) | -3.98 (-11.5 ; 6.16) | 0.136 | 1.02 (0.98 ; 1.05) | 0.388 |
| **P_(a-et)_CO_2_/PaCO_2_ (%)** | -9.19 (-23.4 ; 4.1) | 1.41 (-44.3 ; 24.2) | 0.438 | NIR |  |
| **V_E_/VCO_2-min_** | 2.19 (-9.83 ; 17.49) | 5.97 (-0.08 ; 28.1) | 0.101 | NIR |  |

Values are Δchange in % (95% Confidence interval of Δchange). NIR : Not included In Regression. Hosmer and Lemeshow Test Chi2 (8 df) = 8.37 ; p = 0.398

**Table S12. Multivariate and multivariate analysis of responding parameters during PP session and after PP session, regarding ICU death.**

|  | **Dead** | **Survivors** | **p-univariate** | **Odds Ratio** | **p-multivariate** |
| --- | --- | --- | --- | --- | --- |
| **Before PP vs before SP** | | | | | |
| **ΔpH > 0** | 27 (75) | 56 (88.9) | 0.091 | 0.78 (0.16 ; 3.76) | 0.76 |
| **ΔPaCO_2_ < 0** | 26 (72.2) | 45 (71.4) | 1 | NIR |  |
| **ΔBicarbonates > 0** | 20 (55.6) | 43 (68.3) | 0.278 | NIR |  |
| **ΔFiO_2_ < 0** | 35 (92.1) | 60 (93.8) | 1 | NIR |  |
| **ΔP_plat_ < 0** | 10 (50) | 31 (67.4) | 0.269 | NIR |  |
| **ΔC_stat_ > 0** | 16 (80) | 32 (69.6) | 0.549 | NIR |  |
| **ΔS_III_ < 0** | 14 (48.3) | 34 (57.6) | 0.496 | NIR |  |
| **ΔPetCO_2_ < 0** | 22 (62.9) | 43 (68.3) | 0.658 | NIR |  |
| **ΔPaO2/FiO2 > 0** | 30 (83.3) | 61 (96.8) | 0.026 | 1.04 (0.09 ; 11.9) | 0.975 |
| **ΔΔP < 0** | 15 (75) | 30 (65.2) | 0.569 | NIR |  |
| **ΔV_D_/V_T-phy_ < 0** | 12 (41.4) | 29 (49.2) | 0.507 | NIR |  |
| **ΔP_(a-et)_CO_2_/PaCO_2_ < 0** | 21 (63.6) | 37 (62.7) | 1 | NIR |  |
| **Before PP vs after SP** | | | | | |
| **ΔpH > 0** | 24 (66.7) | 56 (88.9) | 0.015 | 0.23 (0.05 ; 1.05) | 0.058 |
| **ΔPaCO_2_ < 0** | 26 (72.2) | 49 (77.8) | 0.628 | NIR |  |
| **ΔBicarbonates > 0** | 16 (44.4) | 36 (57.1) | 0.296 | NIR |  |
| **ΔFiO_2_ < 0** | 29 (76.3) | 55 (85.9) | 0.284 | NIR |  |
| **ΔP_plat_ < 0** | 12 (54.5) | 28 (60.9) | 0.793 | NIR |  |
| **ΔC_stat_ > 0** | 16 (72.7) | 32 (69.6) | 1 | NIR |  |
| **ΔS_III_ < 0** | 18 (62.1) | 42 (70) | 0.478 | NIR |  |
| **ΔPetCO_2_ < 0** | 27 (77.1) | 49 (76.6) | 1 | NIR |  |
| **ΔPaO2/FiO2 > 0** | 27 (75) | 55 (87.3) | 0.166 | 0.54 (0.13 ; 2.27) | 0.397 |
| **ΔΔP < 0** | 15 (68.2) | 27 (58.7) | 0.595 | NIR |  |
| **ΔV_D_/V_T-phy_ < 0** | 16 (55.2) | 37 (61.7) | 0.647 | NIR |  |
| **ΔP_(a-et)_CO_2_/PaCO_2_ < 0** | 20 (69) | 28 (49.1) | 0.109 | 2.81 (0.99 ; 7.94) | 0.051 |

Values are n (%) and Odds Ratio (95% Confidence interval of OR). NIR: not included in regression. Δchange > 0 or < 0 has been assessed for average PP session for each patient. Parameters with p-value < 0.16 were included in multivariate analysis. Hosmer and Lemeshow test chi^2^ (3df) = 3.727; p = 0.293.

**Table S13. Univariate analysis of Δ**change, before SP-2 hours after SP, in respiratory parameters between dead and survivors during the first PP session.

| **Parameters** | **Dead** | **Survivors** | **P-value** |
| --- | --- | --- | --- |
| **pH** | 0.07 (-0.62 ; 0.44) | 0 (-0.41 ; 0.54) | 0.976 |
| **PaO_2_ (mmHg)** | -5.73 (-18.5 ; 11.6) | -8.25 (-20.5 ; 11.8) | 0.843 |
| **PaCO_2_ (mmHg)** | -2.25 (-9.11 ; 10.6) | -2.38 (-13.9 ; 6) | 0.322 |
| **Bicarbonates (mmol/L)** | -4.37 (-6.79 ; 2.7) | -4.22 (-8.33 ; 1.33) | 0.774 |
| **SaO_2_ (%)** | -0.91 (-2.95 ; 1.9) | -0.4 (-2.41 ; 1) | 0.787 |
| **FiO_2_ (%)** | 15.5 (0 ; 33.3) | 14.3 (0 ; 38.8) | 0.822 |
| **P_moy_ (cmH_2_O)** | 0 (0 ; 4.89) | 0 (-4.65 ; 4.65) | 0.659 |
| **P_plat_ (cmH_2_O)** | 1.75 (-2.14 ; 7.15) | 0 (-3.85 ; 4.76) | 0.244 |
| **PEEP_tot_ (cmH_2_O)** | 0 (0 ; 8.21) | 0 (-0.62 ; 5.76) | 0.517 |
| **RR (n/min)** | 0 (0 ; 0) | 0 (0 ; 0) | 0.894 |
| **VT (mL/kg IBW)** | 0.52 (-2.2 ; 5.27) | 0 (-5.45 ; 1.83) | 0.165 |
| **C_stat_ (mL/cmH_2_O)** | -1.52 (-15.6 ; 4.74) | -1.92 (-15.2 ; 13.3) | 0.6 |
| **C_dyn_ (mL/cmH_2_O)** | -4.71 (-19 ; 7.05) | -4.12 (-14.7 ; 5.14) | 0.802 |
| **ΔP_dyn_ (cmH_2_O)** | 1.02 (-6.74 ; 22.5) | 2.03 (-5.61 ; 13.9) | 0.718 |
| **R_aw_ (cmH_2_O/L/s)** | -10.6 (-30 ; 17) | 0 (-9.32 ; 14.3) | 0.141 |
| **Mechanical Power (J/min)** | -7.15 (-14.1 ; 7.82) | 1.56 (-15 ; 11.3) | 0.588 |
| **P_max_ (cmH_2_O)** | 0 (-4.04 ; 7.05) | 0 (-3.48 ; 7.69) | 0.852 |
| **Vds (mL)** | -6.86 (-11.1 ; 1.03) | -5.63 (-12 ; 1.98) | 0.891 |
| **S_III_ (%CO_2_/L)** | -16.3 (-32.1 ; 5.01) | -12.9 (-24.6 ; 11.8) | 0.388 |
| **PetCO_2_ (mmHg)** | -6.67 (-12.8 ; 0) | -3.33 (-10.7 ; 2.79) | 0.313 |
| **FeCO_2_ (%)** | -5.26 (-12.3 ; 2.81) | -3.57 (-9.09 ; 2.08) | 0.635 |
| **VCO_2-min_ (mL/min)** | -4.48 (-7.24 ; 0.59) | -0.24 (-6.41 ; 3.78) | 0.19 |
| **PaO_2_/FiO_2_** | -19 (-31.1 ; -4.33) | -19.2 (-33.1 ; -4.46) | 0.849 |
| **ΔP (cmH_2_O)** | 0 (-17 ; 12.9) | 0 (-10.8 ; 16.7) | 0.721 |
| **Oxygenation Index** | 22.5 (7.24 ; 43.3) | 24.1 (3.49 ; 55.6) | 0.883 |
| **S_III-R_** | -16.5 (-26 ; 3.86) | -11.8 (-24.6 ; 6.96) | 0.541 |
| **Oxygen Stretch Index** | 22.3 (-13 ; 59) | 26.9 (2.39 ; 51.1) | 0.511 |
| **V_D_/V_T-phy_ (%)** | -7.14 (-13.5 ; 4.04) | -4.1 (-12.4 ; 5.78) | 0.729 |
| **P_(a-et)_CO_2_/PaCO_2_ (%)** | 18.9 (-3.19 ; 29.2) | 3.38 (-22.9 ; 38.8)) | 0.599 |
| **V_E_/VCO_2-min_** | 3.41 (-2.43 ; 7.31) | 0.65 (-4.54 ; 6.47) | 0.379 |

Values are Δchange in % (95% Confidence interval of Δchange)

**Table S14. Univariate analysis of Δ**change, before SP-2 hours after SP, in respiratory parameters between dead and survivors during the average PP session per patient.

| **Parameters** | **Dead** | **Survivors** | **P-value** |
| --- | --- | --- | --- |
| **pH** | -0.15 (-0.82 ; 0.17) | -0.02 (-0.41 ; 0.41) | 0.206 |
| **PaO_2_ (mmHg)** | -4.8 (-18.4 ; 6.6) | -6.05 (-19.4 ; 8.66) | 0.916 |
| **PaCO_2_ (mmHg)** | 1.23 (-8.65 ; 10.3) | -2.38 (-10.9 ; 6.7) | 0.169 |
| **Bicarbonates (mmol/L)** | -3.69 (-6.55 ; 0.67) | -2.04 (-5.24 ; 1.48) | 0.282 |
| **SaO_2_ (%)** | -0.78 (-2.39 ; 0.8) | -0.65 (-1.96 ; 0.58) | 0.992 |
| **FiO_2_ (%)** | 21.7 (0 ; 36.3) | 13.9 (0 ; 32.8) | 0.467 |
| **P_moy_ (cmH_2_O)** | 0 (-2.92 ; 3.02) | 0 (-2.75 ; 4.82) | 0.669 |
| **P_plat_ (cmH_2_O)** | 2.73 (-3.47 ; 7.5) | 0.33 (-3.57 ; 4.65) | 0.53 |
| **PEEP_tot_ (cmH_2_O)** | 0 (-1.04 ; 7.87) | 0 (-0.28 ; 5.58) | 0.852 |
| **RR (n/min)** | 0 (-3.73 ; 0) | 0 (-1.9 ; 0.22) | 0.563 |
| **VT (mL/kg IBW)** | 0 (-4.15 ; 5.08) | -0.05 (-3.8 ; 2.51) | 0.487 |
| **C_stat_ (mL/cmH_2_O)** | -7.04 (-15.7 ; 4.93) | 0.31 (-9.6 ; 10.7) | 0.273 |
| **C_dyn_ (mL/cmH_2_O)** | -1.49 (-19 ; 11.3) | -3.34 (-13.5 ; 5.78) | 0.931 |
| **ΔP_dyn_ (cmH_2_O)** | 1.87 (-6.74 ; 22.5) | 3.79 (-6.62 ; 13.8) | 0.762 |
| **R_aw_ (cmH_2_O/L/s)** | -6.71 (-19.7 ; 12.5) | 0 (-11.9 ; 14.6) | 0.244 |
| **Mechanical Power (J/min)** | -7.96 (-14.2 ; 8.29) | 1.18 (-10.8 ; 9.87) | 0.269 |
| **P_max_ (cmH_2_O)** | 0 (-5.19 ; 5.68) | 0.78 (-2.95 ; 7.93) | 0.481 |
| **Vds (mL)** | -4.88 (-9.38 ; 0.73) | -4.47 (-10.8 ; 2.86) | 0.736 |
| **S_III_ (%CO_2_/L)** | -7.73 (-24.5 ; 7.78) | -9.1 (-23.5 ; 8.42) | 0.835 |
| **PetCO_2_ (mmHg)** | -5.13 (-10.3 ; 1.57) | -3.4 (-9.35 ; 2.82) | 0.714 |
| **FeCO_2_ (%)** | -2.02 (-6.15 ; 2.9) | -2.26 (-8.29 ; 1.97) | 0.629 |
| **VCO_2-min_ (mL/min)** | -1.38 (-5.94 ; 1.85) | -3.05 (-6.98 ; 3.47) | 0.964 |
| **PaO_2_/FiO_2_** | -17.9 (-32.5 ; -9.62) | -17 (-28.9 ; -5.17) | 0.35 |
| **ΔP (cmH_2_O)** | -3.79 (-6.17 ; 13.4) | -0.63 (-11.4 ; -5.17) | 0.829 |
| **Oxygenation Index** | 27.8 (11.5 ; 41.9) | 19.4 (2.67 ; 45.4) | 0.459 |
| **S_III-R_** | -9.46 (-23.8 ; 10.2) | -10 (-24.1 ; 1.04) | 0.831 |
| **Oxygen Stretch Index** | 36.5 (10.7 ; 55.8) | 20.9 (2.24 ; 47.8) | 0.271 |
| **V_D_/V_T-phy_ (%)** | -5.36 (-9.04 ; 1.75) | -3.74 (-10.3 ; 4.01) | 0.67 |
| **P_(a-et)_CO_2_/PaCO_2_ (%)** | 8.63 (-5.55 ; 27.3) | 5.1 (-14.2 ; 54.8) | 0.826 |
| **V_E_/VCO_2-min_** | 0.89 (-8.79 ; 5.04) | 1.44 (-4.84 ; 12.3) | 0.358 |

Values are Δchange in % (95% Confidence interval of Δchange)

**Figure S1. Evolution of ΔV_D_/V_T-phy_, ΔS_III_, ΔP_et_CO_2_ and ΔC_dyn_ for each parameter (all sessions), from 0 hour (just before prone positioning) to 24 hours of prone position; at sessions’ end; 2 hours after return in supine position.**


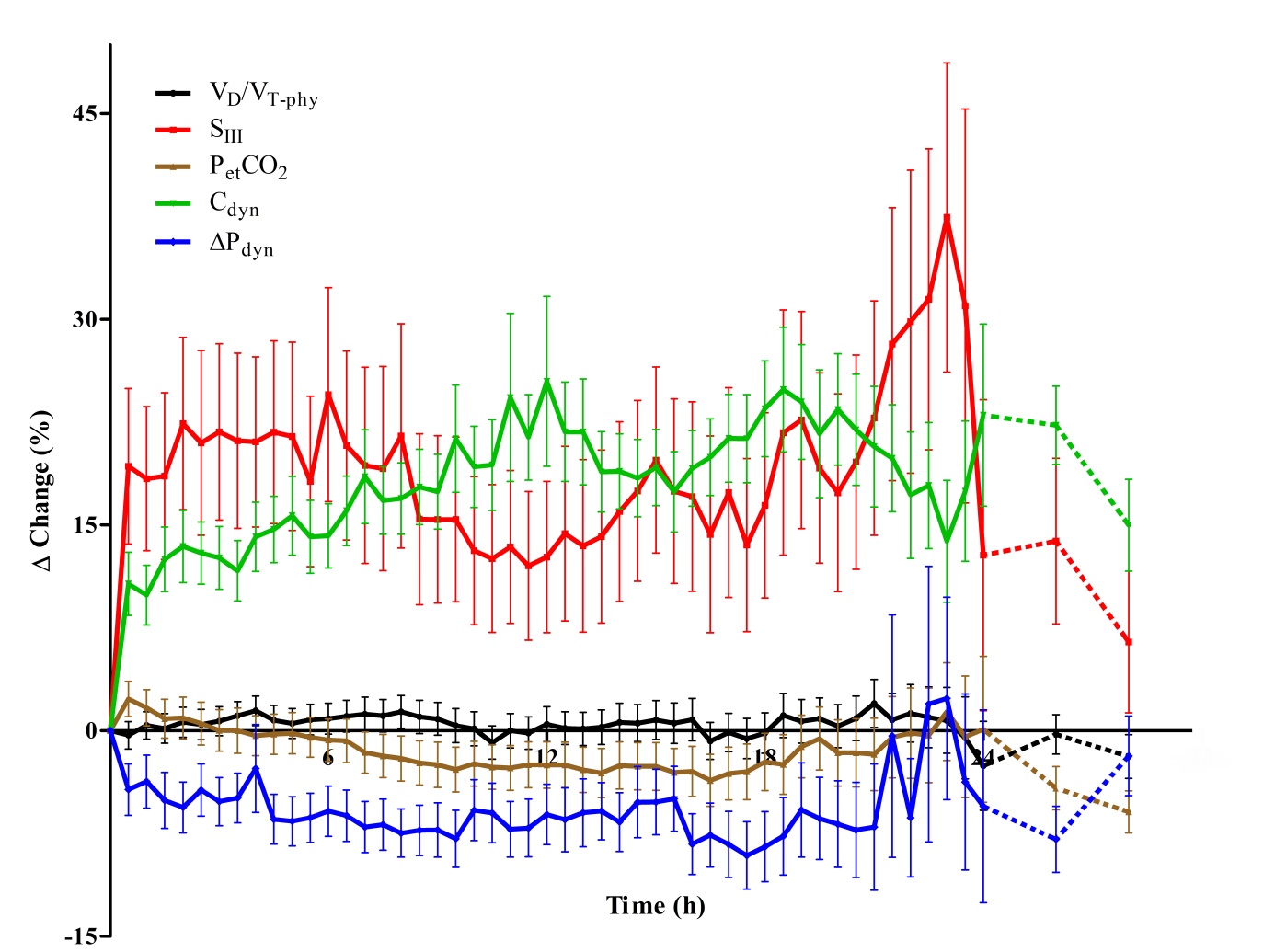


- **Figure S2.** Evolution of ΔV_D_/V_T-phy_, ΔS_III_, ΔP_et_CO_2_ and ΔC_dyn_ for each parameter (all non-responders sessions), from 0 hour (just before prone positioning) to 24 hours of prone position;
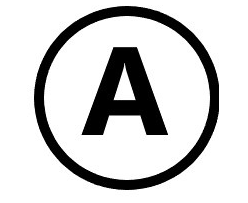
 at sessions’ end;
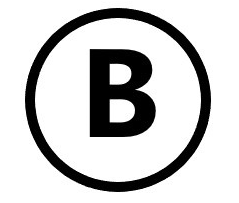
 2 hours after return in supine position. * p < 0.05 (global time effect comparison)

**
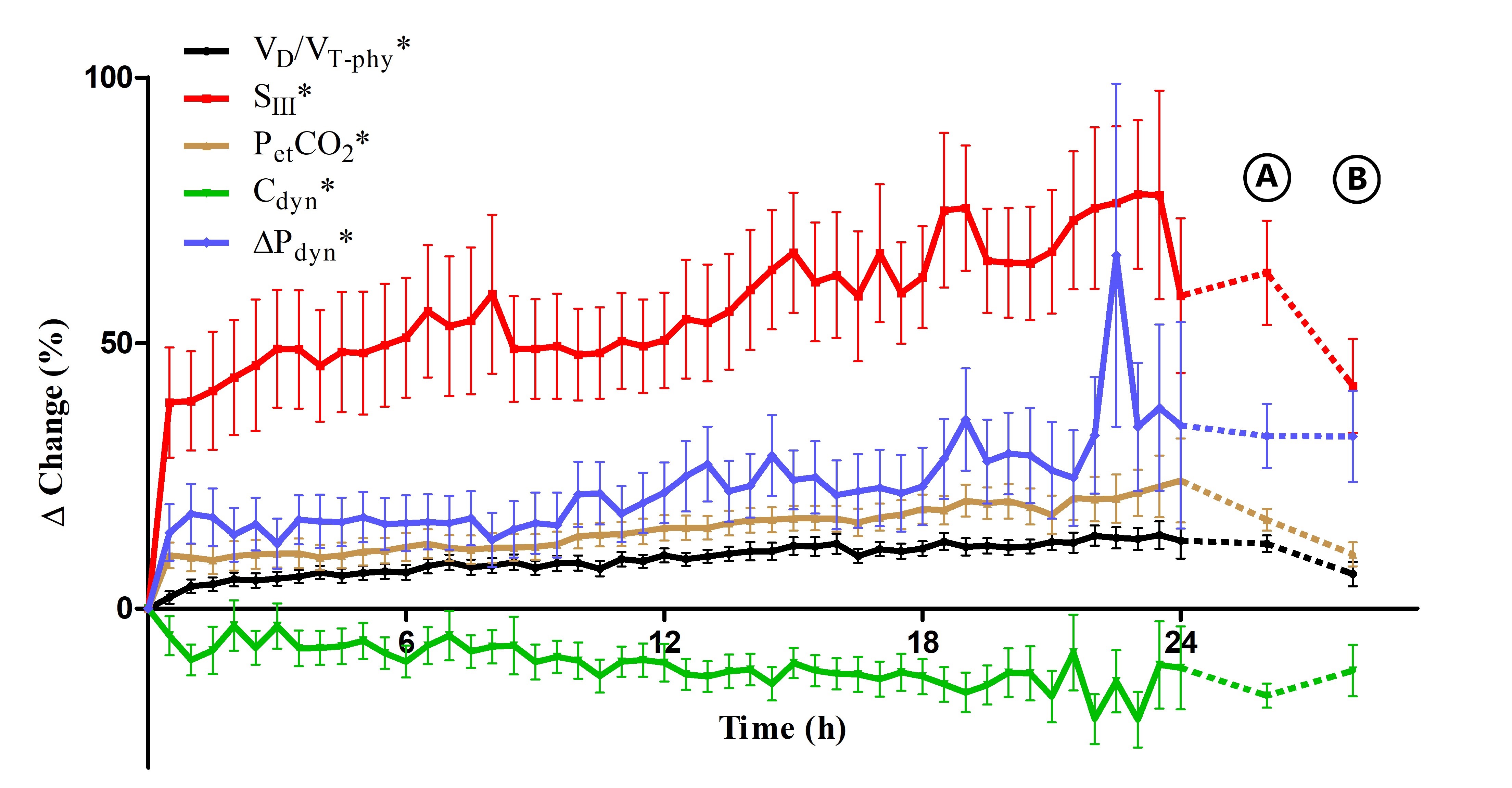
**
